# Urethane Macromonomers: Key Components for the Development of Light-Cured High-Impact Denture Bases

**DOI:** 10.3390/polym17131761

**Published:** 2025-06-26

**Authors:** Benjamin Grob, Pascal Fässler, Iris Lamparth, Sadini Omeragic, Kai Rist, Loïc Vidal, Jacques Lalevée, Yohann Catel

**Affiliations:** 1 Ivoclar Vivadent AG, Bendererstrasse 2, FL-9494 Schaan, Liechtenstein; benjamin.grob@ivoclar.com (B.G.); iris.lamparth@ivoclar.com (I.L.); kai.rist@ivoclar.com (K.R.); 2 Institut de Science des Matériaux de Mulhouse, Université de Haute-Alsace, CNRS, IS2M UMR 7361, 68100 Mulhouse, Francejacques.lalevee@uha.fr (J.L.); 3 Université de Strasbourg, 4 Rue Blaise Pascal, 67000 Strasbourg, France

**Keywords:** denture base, block copolymers, high impact, fracture toughness, 3D printing

## Abstract

The development of high-impact denture base formulations that are suitable for digital light processing (DLP) 3D printing is demanding. Indeed, a combination of high flexural strength/modulus and high fracture toughness is required. In this contribution, eight urethane macromonomers (**UMs1-8**) were synthesized in a one-pot, two-step procedure. Several rigid diols were first reacted with two equivalents of trimethylhexamethylene diisocyanate. The resulting diisocyanates were subsequently end-capped with a free-radically polymerizable monomer bearing a hydroxy group. **UMs1-8** were combined with the monofunctional monomer (octahydro-4,7-methano-1H-indenyl)methyl acrylate and a poly(ε-caprolactone)-polydimethylsiloxane-poly(ε-caprolactone) (PCL-PDMS-PCL) triblock copolymer (**BCP1**) as a toughening agent. The double-bond conversion, glass transition temperature (T_g_), and mechanical properties (flexural strength/modulus, fracture toughness) of corresponding light-cured materials were measured (cured in a mold using a light-curing unit). The results showed that the incorporation of **BCP1** was highly efficient at significantly increasing the fracture toughness, as long as the obtained networks exhibited a low crosslink density. The structure of the urethane macromonomer (nature of the rigid group in the spacer; nature and number of polymerizable groups) was demonstrated to be crucial to reach the desired properties (balance between flexural strength/modulus and fracture toughness). Amongst the evaluated macromonomers, **UM1** and **UM2** were particularly promising. By correctly adjusting the **BCP1** content, light-cured formulations based on those two urethane dimethacrylates were able to fulfill ISO20795-1:2013 standard requirements regarding high-impact materials. These formulations are therefore suitable for the development of 3D printable high-impact denture bases.

## 1. Introduction

Additive manufacturing of dental materials brings several advantages for dentists and dental technicians. Using this technology, personalized materials can be easily prepared following a fast and cost-effective workflow [1,2,3,4,5,6,7,8]. The digital light processing (DLP) technology, which employs a projector as a light source to cure a resin layer by layer, is mainly used for 3D printing of dental materials. 3D printing dental materials including temporary crowns, models, surgical guides, splints, digital dentures (base and teeth materials), and night guards are currently available on the market. 3D printing of high-impact denture bases is particularly challenging. Indeed, such materials must exhibit high flexural strength/modulus and be fracture-tough. Due to improved durability and fracture resistance in case of accidental dropping, high-impact denture bases are superior to conventional denture bases. According to the international ISO20795-1:2013 standard, heat-cured and light-activated denture bases must exhibit flexural strength and modulus values above 65 MPa and 2000 MPa, respectively (measured in water at 37 °C) [9]. Additionally, particularly high maximum stress intensity factor (K_max_ ≥ 1.9 MPa m^1/2^) and total fracture work (W_f_ ≥ 900 J m^−2^) values must be reached for high-impact materials [9].

Following the traditional workflow, complete dentures are manufactured using compression and injection molding techniques [10]. Heat-cured denture bases are typically used for this workflow. These materials mostly contain crosslinked poly(methyl methacrylate) (PMMA). They are commonly obtained by thermal polymerization of a methyl methacrylate (MMA)-based resin, which is prepared by mixing a liquid (containing MMA and low amounts of dimethacrylates), and a powder (mainly containing beads of PMMA and a thermal initiator such as benzoyl peroxide) [10,11]. Contrary to conventional denture bases, high-impact materials additionally contain toughening agents (e.g., core–shell particles). The toughening of thermoplastic polymers such as PMMA with core–shell particles is highly efficient and leads to excellent fracture toughness. Unfortunately, this technology cannot be easily transferred to DLP 3D printing. Indeed, MMA is a volatile monomer and presents an unsuitable reactivity. For these reasons, mixtures of di(meth)acrylates are typically used for 3D printing of denture bases. As a result of their high crosslink density, printed di(meth)acrylate materials are brittle. Geiger et al. showed that these materials exhibit rather low fracture toughness [12]. Moreover, it has been clearly demonstrated that well-known toughening strategies, such as the incorporation of block copolymers (BCPs) or core–shell particles, are quite inefficient for high crosslink density di(meth)acrylate networks [13,14,15]. Indeed, only a moderate fracture toughness increase was observed upon addition of these toughening agents. To the best of our knowledge, no commercially available 3D printing denture bases fulfill the ISO20795-1:2013 standard requirements regarding high-impact materials [9]. There is consequently a strong need for efficient toughening technologies that would be suitable for 3D printing of (meth)acrylate based materials.

**Figure 1:**
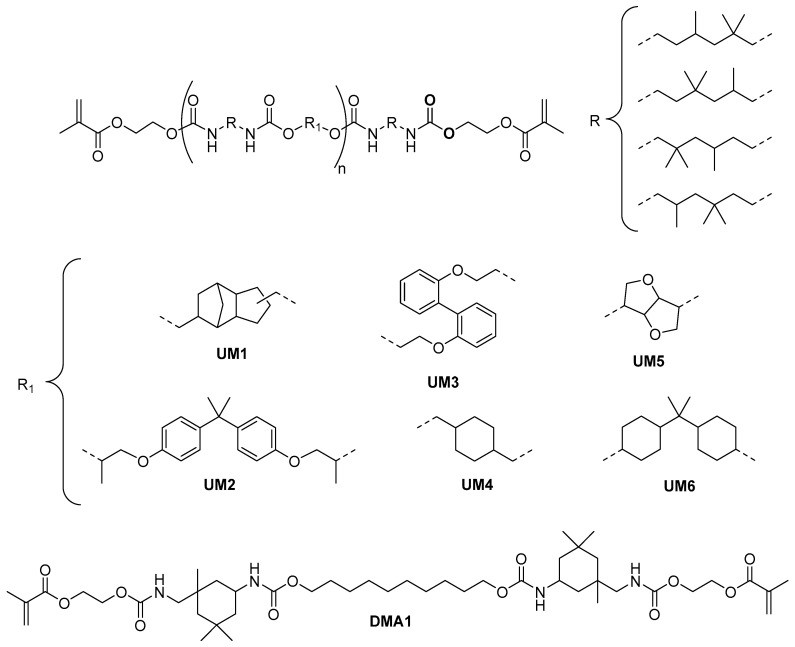
Structures of **DMA1** and **UMs1-6**.

**Figure 2:**
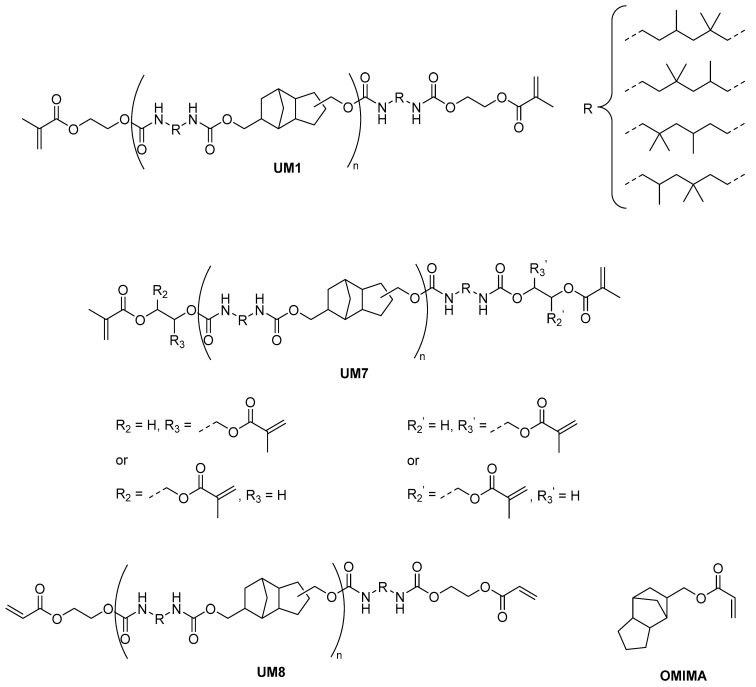
Structures of **UM1**, **UM7**, **UM8**, and OMIMA.

In this context, we recently proposed an approach based on the addition of BCPs to low crosslink density di(meth)acrylate-based networks [16,17,18,19,20]. A poly(ε-caprolactone)-polydimethylsiloxane-poly(ε-caprolactone) (PCL-PDMS-PCL) triblock copolymer was typically added to a monofunctional (meth)acrylate/urethane dimethacrylate macromonomer mixture. Using this strategy, materials presenting excellent flexural strength/modulus and high fracture toughness values were successfully prepared. These resins met the requirements for DLP 3D printing (non-volatile monomers, high reactivity, low viscosity, etc.). It was demonstrated that the structure of the urethane dimethacrylate macromonomer strongly influences the flexural strength/modulus and the fracture toughness of the cured materials [17,19]. The presence of multiple urethane moieties results in the formation of strong hydrogen bonds that beneficially affect the mechanical properties of the network. Most of the urethane macromonomers that we have evaluated so far were synthesized via the reaction of isophorone diisocyanate (IPDI, 2.0 eq.) with a diol (1.0 eq.), followed by an end-capping with 2-hydroxyethyl methacrylate. Longer spacer lengths as well as the use of a flexible diol were demonstrated to be advantageous in order to obtain high fracture toughness. Unfortunately, materials exhibiting higher fracture toughness often presented lower flexural strength/modulus. Using IPDI-based macromonomers, ISO20795-1:2013 standard requirements for high-impact materials could not be fulfilled [9]. Quite recently, we were able for the first time to develop a 3D printable high-impact denture base material [20]. One of the key compounds of this formulation is the promising urethane macromonomer **UM1** (Figure 1). Contrary to the IPDI-based urethane dimethacrylates, **UM1** was prepared via the reaction of a flexible disocyanate (trimethylhexamethylene diisocyanate) with a rigid diol (tricyclo [5.2.1.0(2,6)]decanedimethanol) (similar end-capping, Figure 1). As this strategy seems to be more efficient to find the right balance between fracture toughness and flexural strength/modulus, the objective of this work was to synthesize and evaluate **UM1** derivatives by changing either the nature of the rigid diol or of the end-capping group. In this contribution, seven new urethane macromonomers, **UMs2-8**, were evaluated for the formulation of light-cured high-impact denture bases (Figure 1 and Figure 2). Each macromonomer was combined with the monofunctional monomer (octahydro-4,7-methano-1H-indenyl)methyl acrylate (OMIMA, Figure 2), and a PCL-PDMS-PCL BCP was added as a toughening agent. **BCP1** (PCL-PDMS-PCL: 1000 g mol^−1^–2000 g mol^−1^–1000 g mol^−1^) was selected as it was demonstrated to be highly efficient in combination with **UM1** and OMIMA [20]. In this contribution, the influence of the urethane macromonomer’s nature on the flexural strength, flexural modulus, and fracture toughness of the corresponding photocured materials (cured in a mold using a light-curing unit) is discussed.

## 2. Materials and Methods

### 2.1. Materials

The monomer (octahydro-4,7-methano-1H-indenyl)methyl acrylate (OMIMA) and Dianol 320 HP were provided by Arkema (Colombes, France) and the initiator Genocure TPO by Rahn AG (Zürich, Switzerland). 1,3-Bis(3-aminopropyl)tetramethyldisiloxane, octamethylcyclotetrasiloxane, ε-caprolactone (CL), and tin(II) 2-ethylhexanoate (Sn(EH)_2_) were purchased from abcr GmbH (Karlsruhe, Germany). Tricyclo [5.2.1.0(2,6)]decanedimethanol was purchased from OQ Chemicals GmbH (Monheim, Germany). Trimethylhexamethylene diisocyanate (TMDI; 2,2,4- and 2,4,4-mixture) was provided by Evonik (Essen, Germany) and 2-hydroxyethyl methacrylate (HEMA) by Nordmann Switzerland AG (Zürich, Switzerland). Isosorbide was purchased from Thermo Scientific GmbH (Reinach, Switzerland) and 2,2-bis(4-hydroxycyclohexyl)propane from TCI Europe N.V (Zwijndrecht, Belgium). Glycerol dimethacrylate (GDMA, isomeric mixture) was provided by Evonik (Essen, Germany) and purified by column chromatography before use. As a result, GDMA was obtained as a pure isomeric mixture of glycerol 1,2-dimethacrylate and glycerol 1,3-dimethacrylate (ratio 24/76: mol/mol). All other reagents were purchased from Sigma-Aldrich (Darmstadt, Germany) and were used without further purification. 2,2’-([1,1’-biphenyl]-2,2’-diylbis(oxy))bis(ethan-1-ol) was synthesized according to the literature [21]. **DMA1** and **UM1** were prepared according to a procedure reported in the literature [16,20]. The synthesis of **BCP1** is described in a previous article [20]. Column chromatography was conducted on silica gel (FlashPure ID Silica, particle size: 35–45 μm) using Pure C-815 Flash from BÜCHI Labortechnik AG (Flawil, Switzerland).

### 2.2. Synthesis of Urethane Macromonomers ***UMs2-8***

#### 2.2.1. **UM2**

General procedure: A mixture of Dianol 320 HP (671.92 g, 1.95 mol) and dibutyltin dilaurate (1.19 g, 1.88 mmol) was heated to 40 °C. TMDI (820.39 g, 3.90 mol) was added, and the reaction mixture was stirred at 100 °C for 1 h. Subsequently, BHT (0.37 g, 1.7 mmol) and HEMA (515.35 g, 3.96 mol) were added, and the resulting mixture was stirred for 1 h, affording the desired product in quantitative yield.

Aspect: colorless viscous resin.

^1^H NMR (CDCl_3_, 400 MHz): δ = 0.80–0.99 (m, 18H; CH_3_); 1.00–1.76 (m, 22H; CH, CH_2_, CH_3_); 1.95 (s, 6H; CH_3_); 2.75–3.28 (m, 8H; NCH_2_); 3.85–4.09 (m, 4H; OCH_2_); 4.10–4.41 (m, 8H; OCH_2_); 4.43–5.29 (m, 6H; CHO, NH); 5.58 (s, 2H; =CH); 6.13 (s, 2H; =CH); 6.71–6.86 (m, 4H, ArH), 7.03–7.18 (m, 4H, ArH).

#### 2.2.2. **UM3**

According to the procedure described for **UM1**, **UM3** was prepared from 2,2’-([1,1’-biphenyl]-2,2’-diylbis(oxy))bis(ethan-1-ol) (30.00 g, 109.4 mmol), dibutyltin dilaurate (0.06 g, 0.10 mmol), TMDI (45.99 g, 218.7 mmol), and HEMA (28.46 g, 218.7 mmol), affording **UM3** in quantitative yield.

Aspect: colorless viscous resin.

^1^H NMR (CDCl_3_, 400 MHz): δ = 0.80–0.98 (m, 18H; CH_3_); 1.00–1.78 (m, 10H; CH, CH_2_); 1.91–1.98 (m, 6H; CH_3_); 2.71–3.31 (m, 8H; NCH_2_); 3.98–4.44 (m, 16H; OCH_2_); 4.45–5.48 (m, 4H; NH); 5.51–5.64 (m, 2H; =CH); 6.08–6.18 (m, 2H; =CH); 6.87–7.06 (m, 4H, ArH), 7.14–7.37 (m, 4H, ArH).

#### 2.2.3. **UM4**

According to the procedure described for **UM1**, **UM4** was prepared from cyclohexane-1,4-diyldimethanol (80.00 g, 554.7 mmol), dibutyltin dilaurate (0.292 g, 0.46 mmol), TMDI (233.30 g, 1.11 mol), and HEMA (148.00 g, 1.14 mol), affording **UM4** in quantitative yield.

Aspect: colorless resin.

^1^H NMR (CDCl_3_, 400 MHz): δ = 0.81–0.97 (m, 18H; CH_3_); 0.98–1.89 (m, 20H; CH, CH_2_); 1.93–1.97 (m, 6H; CH_3_); 2.77–3.28 (m, 8H; NCH_2_); 3.82–4.05 (m, 4H; OCH_2_); 4.19–4.47 (m, 8H; OCH_2_); 4.47–5.20 (m, 4H; NH); 5.57–5.62 (m, 2H; =CH); 6.11–6.17 (m, 2H; =CH).

#### 2.2.4. **UM5**

According to the procedure described for **UM1**, **UM5** was prepared from isosorbide (17.67 g, 120.192 mmol), dibutyltin dilaurate (0.06 g, 0.10 mmol), TMDI (50.85 g, 241.84 mmol), and HEMA (31.47 g, 241.84 mol), affording **UM5** in quantitative yield.

Aspect: colorless resin.

^1^H NMR (CDCl_3_, 400 MHz): δ = 0.80–0.98 (m, 18H; CH_3_); 0.99–1.77 (m, 10H; CH, CH_2_); 1.88–1.99 (m, 6H; CH_3_); 2.79–3.29 (m, 8H; NCH_2_); 3.62–4.09 (m, 4H; OCH_2_); 4.25–4.43 (m, 8H; OCH_2_); 4.44–5.29 (m, 8H; NH, OCH); 5.55–5.64 (m, 2H; =CH); 6.09–6.19 (m, 2H; =CH).

#### 2.2.5. **UM6**

According to the procedure described for **UM1**, **UM6** was prepared from 2,2-bis(4-hydroxycyclohexyl)propane (39.14 g, 162.83 mmol), dibutyltin dilaurate (0.06 g, 0.10 mmol), TMDI (68.48 g, 325.66 mmol), and HEMA (43.02 g, 330.54 mmol), affording **UM6** in quantitative yield.

Aspect: colorless viscous resin.

^1^H NMR (CDCl_3_, 400 MHz): δ = 0.67–0.80 (m, 6H; CH_3_); 0.82–0.99 (m, 18H; CH_3_); 1.00–1.83 (m, 24H; CH, CH_2_); 1.86–2.15 (m, 10H; CH_2_, CH_3_); 2.75–3.29 (m, 8H; NCH_2_); 4.24–4.42 (m, 8H; OCH_2_); 4.43–5.15 (m, 6H; NH, OCH); 5.55–5.64 (m, 2H; =CH); 6.09–6.19 (m, 2H; =CH).

#### 2.2.6. **UM7**

According to the procedure described for **UM1**, **UM7** was prepared from tricyclo [5.2.1.0(2,6)]decanedimethanol (11.65 g, 59.37 mmol), dibutyltin dilaurate (0.05 g, 0.08 mmol), TMDI (24.97 g, 118.70 mmol), and glycerol dimethacrylate (GDMA) (27.10 g, 118.70 mol), affording **UM7** in quantitative yield.

Aspect: colorless resin.

^1^H NMR (CDCl_3_, 400 MHz): δ = 0.83–0.98 (m, 18H; CH_3_); 1.00–1.83 (m, 18H; CH, CH_2_); 1.95 (s, 12H; CH_3_); 1.99–2.58 (m, 6H; CH, CH_2_); 2.78–3.26 (m, 8H; NCH_2_); 3.71–4.06 (m, 4H; OCH_2_); 4.17–4.45 (m, 8H; OCH_2_); 4.53–5.19 (m, 4H; NH); 5.20–5.50 (m, 2H, OCH); 5.54–5.66 (m, 4H; =CH); 6.05–6.19 (m, 4H; =CH).

#### 2.2.7. **UM8**

According to the procedure described for **UM1**, **UM8** was prepared from tricyclo [5.2.1.0(2,6)]decanedimethanol (22.38 g, 114.00 mmol), dibutyltin dilaurate (0.06 g, 0.10 mmol), TMDI (47.95 g, 228.00 mol), and 2-hydroxyethyl acrylate (26.46 g, 228.00 mol), affording **UM8** in quantitative yield.

Aspect: colorless resin.

^1^H NMR (CDCl_3_, 400 MHz): δ = 0.83–0.98 (m, 18H; CH_3_); 1.00–1.85 (m, 18H; CH, CH_2_); 1.91–2.58 (m, 6H; CH, CH_2_); 2.78–3.26 (m, 8H; NCH_2_); 3.74–4.00 (m, 4H; OCH_2_); 4.25–4.45 (m, 8H; OCH_2_); 4.51–5.29 (m, 4H; NH); 5.81–5.91 (m, 2H; =CH); 6.08–6.22 (m, 2H; =CH); 6.39–6.50 (m, 2H; =CH).

### 2.3. Formulation of Photopolymerizable BCP-Based Monomer Mixtures

**UMs1-8** and **DMA1** were firstly mixed with the monofunctional monomer OMIMA. **BCP1** and 1.0 wt% TPO were subsequently added. The mixture was stirred at 50 °C until full solubilization of the BCP.

### 2.4. Measurements

#### 2.4.1. Nuclear Magnetic Resonance (NMR) Spectroscopy

NMR measurements were performed on a DPX-400 (Bruker Biospin, Fällanden, Switzerland) in deuterated solvents with tetramethylsilane (TMS) as standard. Data are displayed in the following order: chemical shift in ppm, multiplicity (bs, broad singlet; s, singlet; t, triplet; q, quadruplet; m, multiplet), coupling constant in Hertz (Hz), and assignment.

#### 2.4.2. Fourier Transform Infrared Spectroscopy (FT-IR)

IR spectroscopy was conducted using a Perkin Elmer FT-IR Spectrum Two (UATR Two) spectrometer (8 scans, 4 cm^−1^ resolution, 600–4000 cm^−1^) in ATR mode. The decrease in the absorption of the isocyanate stretching vibration (2270 cm^−1^) was traced for the determination of the isocyanate conversion.

#### 2.4.3. Flexural Strength and Flexural Modulus

Flexural strength was measured according to ISO20795-1:2013 [9]. Specimens (3.3 mm × 10 mm × 64 mm) were prepared using stainless-steel molds (n = 6). The molds were filled with the photopolymerizable monomer mixture and covered with polyester film (50 µm) to avoid oxygen inhibition. After light-curing for 10 min from both sides in a PrograPrint Cure LED curing unit (120 mW cm^−2^ @ 405 nm and 100 mW cm^−2^ @ 465 nm), the specimens were removed from the mold and stored in water at 37 °C for 50 h. Measurement of flexural strength and modulus was carried out in a three-point bending test (span: 50 mm) with a cross-head speed of 5 mm min^−1^ using a Z2.5/TS universal testing machine (ZwickRoell, Ulm, Germany). The measurement was carried out in water at 37 °C (the specimens were immersed in a tempered water bath during the measurement).

#### 2.4.4. Fracture Toughness

Fracture toughness was measured using a modified bending test described in ISO 20795-1:2013 [9]. Both the maximum stress intensity factor (K_max_) and work of fracture (W_f_) were determined using single-edge notched beam (SENB) specimens (n = 6). A stainless-steel mold (4 mm × 8 mm × 40 mm) was filled with a photopolymerizable monomer mixture. The mold was covered with a polyester film (50 µm) to avoid oxygen inhibition. After light-curing for 10 min from both sides in a PrograPrint Cure LED curing unit (120 mW cm^−2^ @ 405 nm and 100 mW cm^−2^ @ 465 nm), cured specimens were removed from the mold and a 3.0 mm deep notch was prepared using a circular saw with diamond blade and the initial crack was prepared by striking a razorblade with gentle pressure to a depth of 0.3 mm. Specimens were stored in water for 24 h at 37 °C, dabbed with paper and subsequently loaded to break at RT with a span of 32 mm and at a crosshead speed of 1.0 mm/min using a universal testing machine (Zwick Z2.5, ZwickRoell, Ulm, Germany).

K_max_ (in MPa m^1/2^) was calculated as follows:

(1) Kmax=f(x)Pmaxlt(btht32)×10−3 MPa m12

where *f*(*x*) is a geometrical function dependent on *x*:

(2) f(x)=3x12[1.99−x(1−x)(2.15−3.93x+2.7x2)]/[2(1+2x)(1−x)32] 

with *x =* (*a*/*h_t_*) and *h_t_* as the height of the specimen (8 mm), *b_t_* its width (4 mm), *l_t_* the span length (32 mm), *a* the crack length (3 mm + crack depth with razor blade), and *P_max_* the maximum load exerted on the specimens (in N).

The total fracture work *W_f_* (in J m^−2^) was calculated as follows:

(3) $$W_{f}=\frac{U}{\left[2b_{t}(h_{t}-a)\right]}\times 1000$$

where *U* is the recorded area under the load/deflection curve.

#### 2.4.5. Viscosity

The viscosity of the photopolymerizable monomer mixtures was measured in a rheometer (MCR302, Anton-Paar, Graz, Austria) at 23 °C with cone-plate setup (CP25-2, 53 µm gap) in rotational mode at a shear rate of 100 s^−1^.

#### 2.4.6. Measurement of T_g_ Using Dynamic Mechanical Thermal Analysis (DMTA)

DMTA specimens were prepared using a stainless-steel mold (5 mm × 2 mm × 40 mm) with polyester film (50 µm) as cover to avoid oxygen inhibition. After light-curing for 10 min from both sides in a PrograPrint Cure LED curing unit (120 mW cm^−2^ @ 405 nm and 100 mW cm^−2^ @ 465 nm), specimens were removed from the mold. DMTA measurements were performed on an Anton Paar MCR 301 device with a CTD 600 oven and a SRF 5 fixture. The measurements were carried out in torsion mode with a frequency of 1 Hz, a normal force (FN) of–1 N, and a strain of 0.05%. A temperature spectrum was monitored from 25 °C to 250 °C with a heating rate of 2 °C min^−1^. The glass transition temperature (T_g_) was defined as the temperature corresponding to the maximum of the loss factor (tan δ) curve.

#### 2.4.7. Near-Infrared (NIR) Spectrometry (Measurement of the Double Bond Conversion)

Circular specimens (d = 15 mm, h = 1 mm, n = 3) were prepared using stainless-steel molds. The molds were filled with the photopolymerizable monomer mixture and covered with a polyester film (50 µm) to avoid oxygen inhibition. After light-curing for 10 min from both sides in a PrograPrint Cure LED curing unit (120 mW cm^−2^ @ 405 nm and 100 mW cm^−2^ @ 465 nm), the specimens were removed from the molds and stored in distilled water at 37 °C for 24 h before the evaluation of the DBC. To obtain the double bond conversion, the spectra of the uncured and cured material were measured by NIR spectroscopy using the Invenio R spectrometer (Bruker, 16 Scans, 8 cm^−1^ Resolution, 3000–10,000 cm^−1^) at a film thickness of 1.0 mm. The (meth)acrylate overtone peak at 6165 cm^−1^ was integrated for both spectra. DBC was calculated by the following equation:

(4) $$DBC=\left(1-\left(\frac{A_{cured}}{A_{uncured}}\right)\right)*100$$

A_cured_ and A_uncured_ correspond to the integrated area in the NIR spectrum (A_cured_: area of the (meth)acrylate peak of the cured material, A_uncured_: area of the (meth)acrylate peak of the uncured material).

#### 2.4.8. Scanning Transmission Electron Microscopy (STEM)

Samples of 70 nm thickness were prepared by ultramicrotomy (Leica model EM UC7) at room temperature and deposited on the surface of high-resolution copper grids. In order to visualize the areas containing block copolymer, the sections were stained at 20 °C for 24 h with osmium tetroxide (OsO_4_) vapor. As the staining was found to be rather selective on the block copolymer, no further treatment of the sections was necessary. It should be noted that the staining of aromatic groups is very low compared with the block copolymer. Observations were made using a JEOL ARM-200F (JEOL, Tokyo, Japan) transmission microscope operating at an accelerating voltage of 200 kV.

## 3. Results

### 3.1. Synthesis of Urethane Macromonomers ***UMs2-8*** and of ***BCP1***

**Figure 3:**
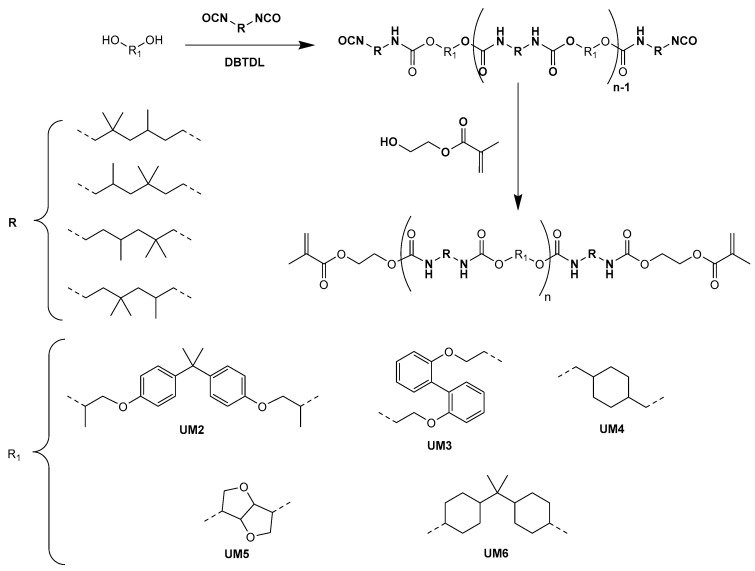
Synthesis of UMs2-6.

For the preparation of **UMs2-6**, a two-step one-pot synthesis was performed (Figure 3). In the first step, the selected diol was reacted with two equivalents of trimethylhexamethylene diisocyanate (TMDI; 2,2,4- and 2,4,4-mixture of isomers) in the presence of a catalytic amount of dibutyltin dilaurate (DBTDL), affording a mixture of urethane oligomers bearing isocyanate end groups. Subsequently, the remaining isocyanate moieties were quenched with 2-hydroxyethyl methacrylate (HEMA). This reaction step was monitored by FT-IR and was stopped after complete consumption of the isocyanate groups. Macromonomers **UMs2-6** were obtained as viscous resins.

**Figure 4:**
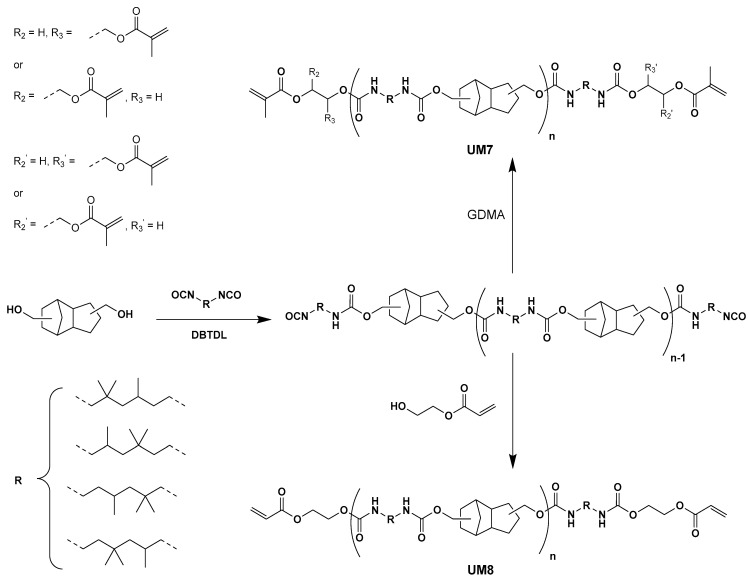
Synthesis of **UM7** and **8**.

**UM7** and **UM8** were prepared according to a similar synthetic pathway (Figure 4). However, 2-hydroxyethyl acrylate (HEA) and glycerol dimethacrylate (GDMA) were used for the end-capping step instead of HEMA. **UMs2-8** were characterized by ^1^H NMR spectroscopy and by gel permeation chromatography (GPC). The NMR spectra and GPC results can be found in the Supporting Information. Owing to the lack of selectivity of the isocyanate moieties, a mixture of urethane oligomers exhibiting different lengths and of bis-[(2-methacryloyloxyethoxy-carbonyl)-amino]-2,2,4-trimethylhexane (UDMA, n = 0) was obtained for each urethane macromonomer synthesis. This behavior was previously observed and described by Fässler et al. for **UM1** [21]. **UMs2-8** were used without further purification for the formulation of **BCP1**-based resins.

**Figure 5:**
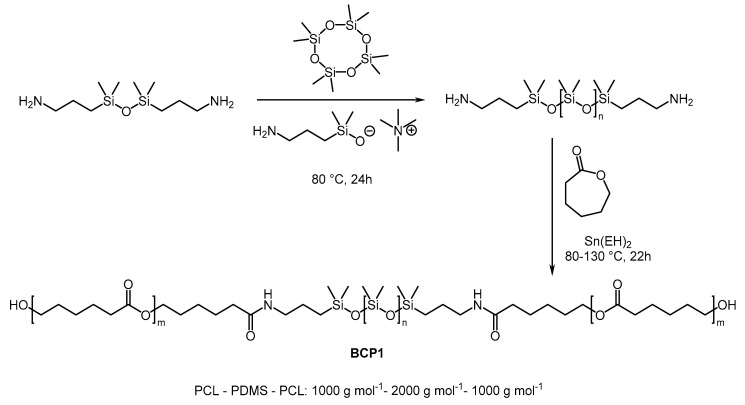
Synthesis of **BCP1**.

**BCP1** was prepared in two steps, according to a procedure described in the literature (Figure 5) [20].

### 3.2. Evaluation of Novel Urethane Dimethacrylate Macromonomers: Variation of the Nature of the Diol

**UMs1-6** were mixed with the monofunctional monomer OMIMA (ratio: 1/1: wt/wt) and **BCP1** (5.0 wt%) was added. **DMA1**, a dimethacrylate that was evaluated in a previous work, was selected as a reference [16]. The viscosity of each formulation was determined (Table 1). The nature of the urethane macromonomer was shown to have a strong influence on this property. Mixtures based on **DMA1** and **UM1** exhibited the lowest viscosity values, whereas the formulation containing **UM6** was the most viscous one. Nevertheless, the viscosity of each formulation was suitable for DLP 3D printing application. Indeed, the viscosity of commercially available DLP 3D printing resins typically lies in the range of 0.5 to 10.0 Pa s. TPO (1.0 wt%) was added as a photoinitiator and each formulation was photocured. DBC and T_g_ were measured. Interestingly, the photocuring of each formulation led to full DBC (see Supporting Information). T_g_ values varied from 72 °C (**UM3**/OMIMA) to 101 °C (**DMA1**/OMIMA) (the corresponding curves can be found in the Supporting Information).

**Figure 6:**
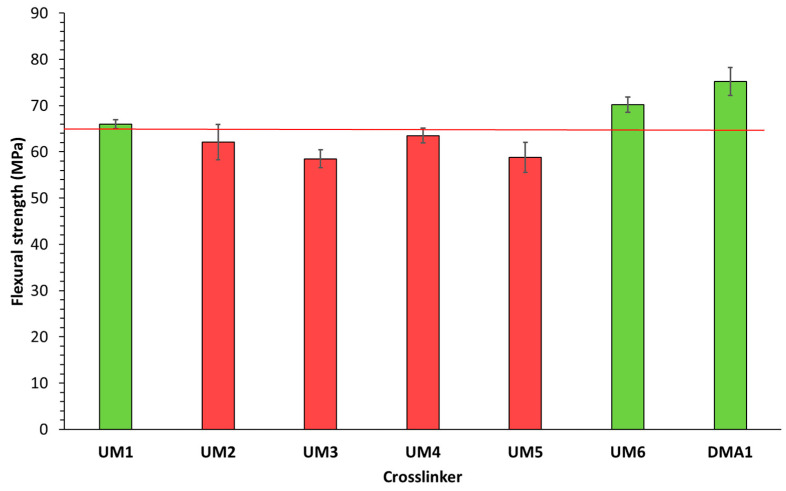
Flexural strength (ISO20795-1:2013) for **UMs1-6**/OMIMA and **DMA1**/OMIMA materials containing 5.0 wt% **BCP1**. The red line represents the minimally required value according to ISO20795-1:2013 [9].

**Figure 7:**
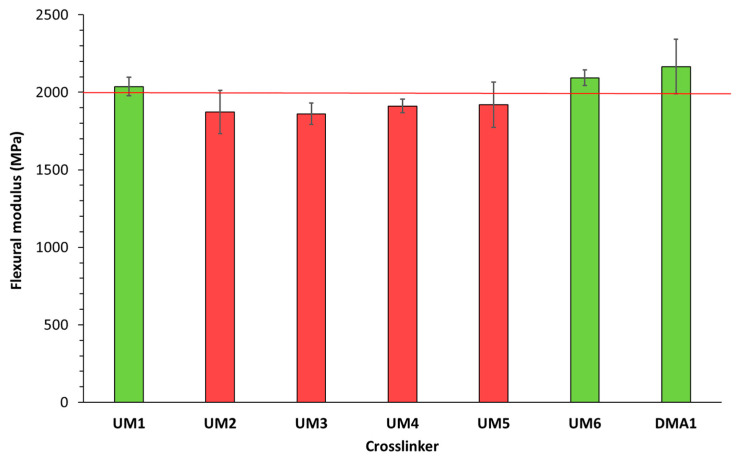
Flexural modulus (ISO20795-1:2013) for **UMs1-6**/OMIMA and **DMA1**/OMIMA materials containing 5.0 wt% **BCP1**. The red line represents the minimally required value according to ISO20795-1:2013 [9].

**Figure 8:**
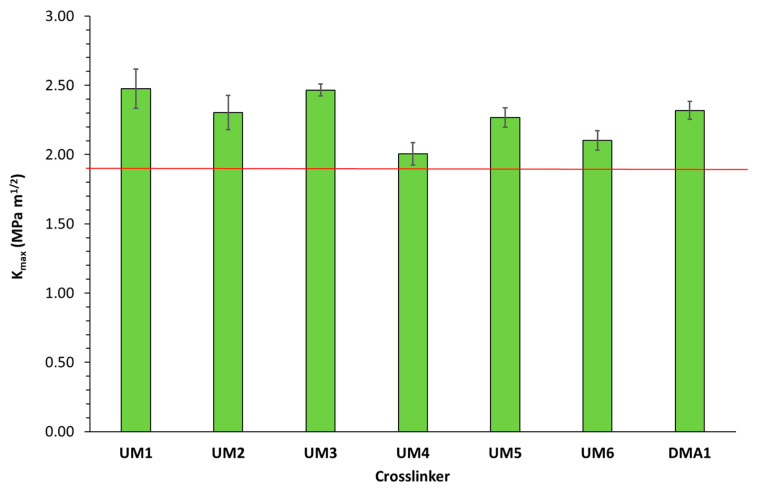
Maximum stress intensity factor (K_max_) for **UMs1-6**/OMIMA and **DMA1**/OMIMA materials containing 5.0 wt% **BCP1**. The red line represents the minimally required value according to ISO20795-1:2013 [9].

**Figure 9:**
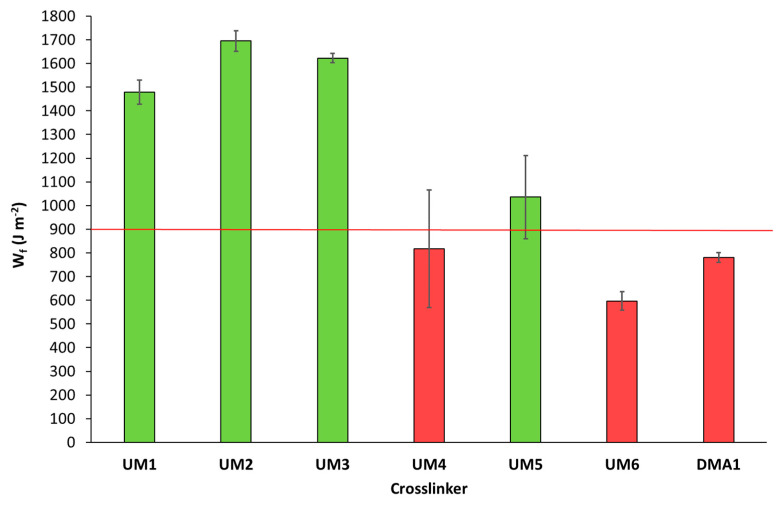
Total fracture work (W_f_) for **UMs1-6**/OMIMA and **DMA1**/OMIMA materials containing 5.0 wt% **BCP1**. The red line represents the minimally required value according to ISO20795-1:2013 [9].

The flexural strength and modulus of these light-cured materials (cured in a mold using a light-curing unit) were subsequently determined (Figure 6 and Figure 7). Results showed that three materials were able to fulfill the ISO20795-1:2013 requirements for high-impact denture base materials: **UM1**/OMIMA (1/1: wt/wt) + 5.0 wt% **BCP1**, **UM6**/OMIMA (1/1: wt/wt) + 5.0 wt% **BCP1**, and **DMA1**/OMIMA (1/1: wt/wt) + 5.0 wt% **BCP1**. **UM2-** and **UM4**-based materials almost reached the desired flexural strength value. The fracture toughness was also measured (Figure 8 and Figure 9). For each material, the maximum stress intensity factor (K_max_) was higher than the minimally required value for high-impact denture bases. On the other hand, the desired total fracture work (W_f_) was not reached for all materials. Indeed, significantly lower values were obtained for materials containing **DMA1**, **UM4**, and **UM6**. As a whole, only the **UM1**-containing formulation was suitable for the preparation of light-cured high-impact denture bases.

It is well-known that the amount of added BCP has a strong influence on both the flexural strength/modulus and the fracture toughness. The formulations based on **UM2**, **UM3**, and **DMA1** almost reached the desired properties. In order to further evaluate the potential of these urethane macromonomers, formulations containing various amounts of **BCP1** were prepared and tested (**UM1**/OMIMA was used as comparison). Results are presented in Table 2 and Table 3. The reduction of the **BCP1** content in **UM1**/OMIMA and **UM2**/OMIMA formulations led to a significant increase in both flexural strength and modulus. Interestingly, it was not the case with **UM3**/OMIMA. Indeed, only a moderate improvement of the mechanical properties was obtained. Regarding the fracture toughness, lower **BCP1** amounts provided lower K_max_ and W_f_ values. Indeed, 4.0 wt% **BCP1** was not sufficient to reach the ISO20795-1:2013 requirements in terms of W_f_ for the **UM1**-based material. On the other hand, this strategy worked for the **UM2**/OMIMA formulation. Indeed, **UM2**/OMIMA + 4.0wt% **BCP1** was also found to be a suitable resin for high-impact denture bases (ISO20795-1:2013 requirements in terms of flexural strength/modulus and fracture toughness are fulfilled). The results obtained with **UM3** were not satisfactory: The addition of 3.0 wt% **BCP1** led to an insufficient toughening effect (W_f_ was too low) combined with unsuitable flexural strength and modulus. Contrary to the **UMs1-3**-containing formulations, the amount of **BCP1** in **DMA1**/OMIMA was increased. As expected, the higher the **BCP1** content, the higher the W_f_ value. Indeed, 7.0wt% **BCP1** was sufficient to exceed the targeted W_f_ value of 900 J m^−2^. However, this material did not meet the ISO20795-1:2013 requirements concerning flexural strength and modulus [9].

**Figure 10:**
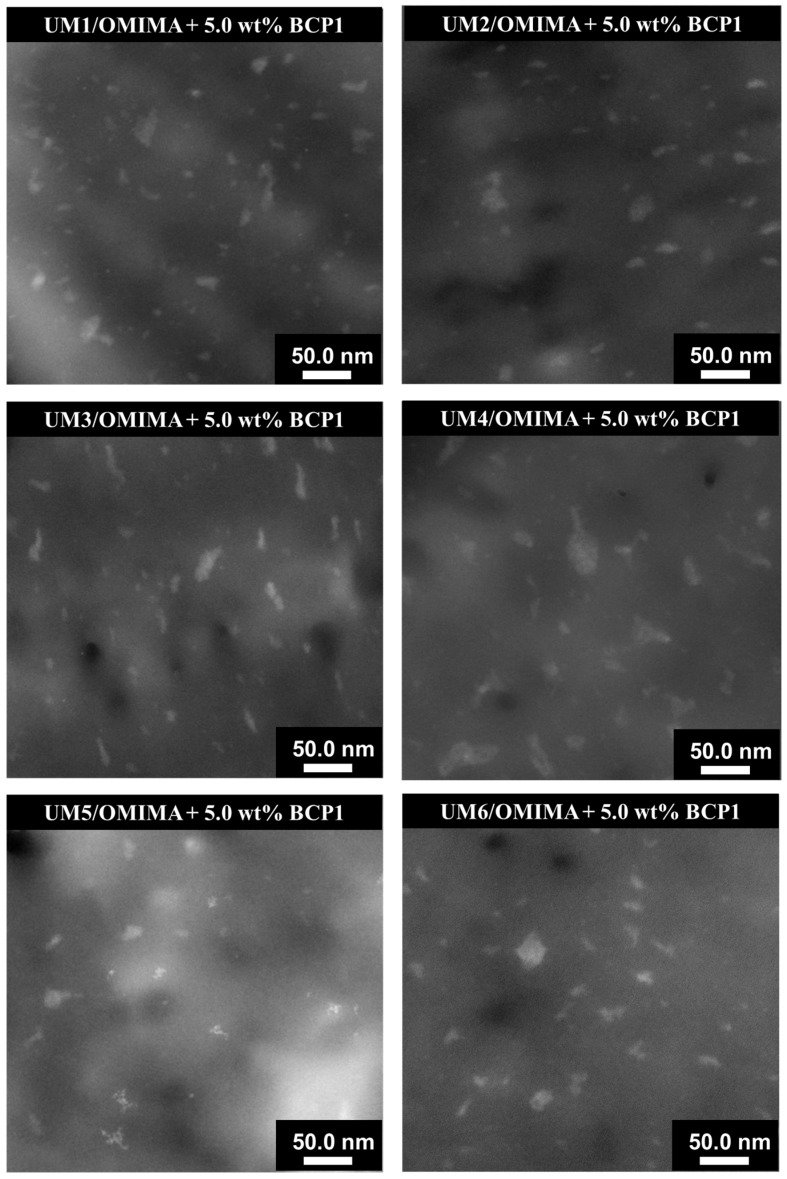
STEM micrographs of cured **UMs1-6**/OMIMA formulations containing 5.0 wt% **BCP1**.

STEM measurements of the materials containing 5.0 wt% **BCP1** were carried out. The STEM micrographs highlighted the presence of well-dispersed nano-objects (Figure 10). No significant differences were observed between the materials.

### 3.3. Evaluation of Novel Urethane Dimethacrylate Macromonomers: Variation of the Nature of the Macromonomer Polymerizable End Groups

**UM1** was shown to be a highly promising urethane macromonomer for the formulation of high-impact materials. The diol used for its synthesis is therefore particularly attractive. For this reason, it was selected for the preparation of **UM7** and **UM8**. The objective was to vary the nature of the polymerizable end group and to evaluate its influence on the mechanical properties of the corresponding cured materials. DBC and T_g_ of photocured **UM7**/OMIMA and **UM8**/OMIMA materials containing 5.0 wt% **BCP1** were assessed (Table 4). It was shown that the T_g_ of the **UM7**-based material was significantly higher than for the other cured resins. **UM8**/OMIMA provided the lowest T_g_ value. Due to the presence of additional methacrylate moieties, light-curing of the **UM7**-containing formulation did not lead to a full DBC (see Supporting Information).

The mechanical properties were assessed (Table 5 and Table 6). High flexural strength and modulus were obtained with the **UM7**-based material. However, extremely low K_max_ and W_f_ values were measured. This material was brittle. The use of a urethane diacrylate macromonomer (**UM8**) instead of a dimethacrylate (**UM1**) was also not advantageous: A decrease in flexural strength/modulus, K_max_, and W_f_ was clearly observed.

## 4. Discussion

The development of 3D printing of high-impact denture bases is highly attractive. However, the formulation of materials exhibiting high flexural strength/modulus as well as high fracture toughness is challenging. Moreover, such formulations must meet specific requirements in order to be suitable for DLP 3D printing: low viscosity, non-volatile monomers, high reactivity, etc. For this reason, the well-known PMMA-based fracture-tough materials are not appropriate for DLP 3D printing. Quite recently, we identified an efficient technology for the preparation of fracture-tough photocurable materials. This technology consists in the incorporation of a block copolymer to a mixture containing a urethane dimethacrylate macromonomer and a monofunctional monomer [16,17,18,19,20]. Our approach was inspired by the pioneering work of Bates, F. S. et al. and by the additional contributions of other research groups regarding the toughening of epoxy materials using BCPs [22,23,24,25,26,27,28,29,30,31,32]. The high efficiency of diblock and triblock copolymers significantly increasing the fracture toughness of crosslinked epoxy materials was indeed clearly demonstrated. Each component of the methacrylate-based formulations has a strong influence on the mechanical properties (flexural strength/modulus and fracture toughness). The challenge in the quest for 3D-printable high-impact denture bases consists in finding the right balance between flexural strength and fracture toughness. Indeed, the addition of BCP as a toughening agent typically leads to a strong increase in the fracture toughness (as long as the crosslink density remains quite low) as well as to a decrease in both flexural strength and modulus. Although it would be quite straightforward to obtain high fracture toughness values via the addition of a BCP to a flexible material, the required flexural strength and modulus would not be reached. A wide range of urethane macromonomers and monofunctional monomers were recently evaluated in formulations containing PCL-PDMS-PCL triblock copolymers [16,17,18,19,20]. For the first time, it was reported that the macromonomer **UM1**, combined with OMIMA, was efficient for the formulation of high-impact denture bases [20]. The use of a flexible diisocyanate (TMDI) combined with a rigid diol for the synthesis of **UM1** is probably paramount for the efficiency of this macromonomer. In this contribution, various urethane macromonomers were synthesized according to the same strategy that was developed for **UM1**: several rigid diols were reacted with an excess of TMDI and the resulting diisocyanate was end-capped with HEMA. **UMs2-6** were efficiently prepared according to this pathway. Resins containing these urethane macromonomers, combined with OMIMA and a PCL-PDMS-PCL BCP, were then evaluated for the development of photocurable high-impact dental bases. The viscosity of these formulations was perfectly suitable for DLP 3D printing. Moreover, full DBC was measured for each cured material. This can be attributed to the high amount of monofunctional monomer in the formulations. This property is highly attractive, as it will prevent any leaching of unreacted monomer from the cured materials. STEM results clearly showed that the incorporation of **BCP1** into the **UMs1-6**/OMIMA mixtures led to the formation of nano-objects. The PCL block is miscible in the monomer mixture, whereas the PDMS middle block is immiscible. It was demonstrated that, in the case of **UM1**/OMIMA, **BCP1** efficiently self-assembles in the uncured mixture, leading thereby to the observed nano-objects in the cured samples [20]. It is highly probable that the nature of the synthesized urethane macromonomers did not strongly affect this phenomenon, and that the nano-objects observed for **UMs2-6**-based materials are also formed via self-assembly. As hypothesized, the nature of the urethane macromonomer structure was demonstrated to have a strong influence on the mechanical properties of the cured materials (light-cured in a mold using a light-curing unit). By correctly adapting the **BCP1** amount in the formulations, **UM2** was also identified as a suitable macromonomer for the preparation of high-impact materials. Indeed, the ISO20795-1:2013 requirements were fulfilled with the **UM2**/OMIMA (1/1: wt/wt) + 4.0 wt% **BCP1** formulation [9]. **UM2** was particularly interesting, as the measured flexural strength/modulus values were significantly above the lower limits that are given by the ISO20795-1:2013 standard. On the other hand, **UMs3-6** did not provide the required properties. The fracture toughness increase obtained with **UM4**- and **UM6**-based materials was not high enough. This result might be due to the specific conformation of the cyclohexyl rings. Excellent fracture toughness values were measured using **UM3**- and **UM5**-containing formulations. Unfortunately, the flexural strength and modulus were not high enough. These macromonomers are therefore too flexible for the aimed application. The comparison of **UMs1-6** with **DMA1** is worth discussing. **DMA1** was identified as a promising urethane dimethacrylate for the preparation of fracture-tough materials in a previous work [16]. Contrary to **UMs1-6**, **DMA1** was synthesized via the reaction of a rigid diisocyanate (IPDI) with a flexible diol. This structure therefore also presents a balance between rigidity and flexibility. The material **DMA1**/OMIMA (1/1: wt/wt) + 5.0 wt% **BCP1** exhibited flexural strength and modulus above the minimally required values for high-impact denture bases. However, K_max_ and W_f_ were too low. The amount of **BCP1** was then increased until the desired fracture toughness was obtained. Such property was reached with 7.0 wt% **BCP1**. Unfortunately, in these conditions, the flexural strength and modulus were lower than the aimed 65 MPa and 2000 MPa, respectively. Therefore, the strategy using TMDI and a rigid diol seems more efficient for the macromonomer synthesis.

Two additional **UM1** alternatives were also considered in this study. **UM7** and **UM8** were prepared by modifying the nature of the polymerizable end-group. **UM8** is a diacrylate monomer, whereas **UM7** bears four methacrylate moieties. The **UM7**/OMIMA + 5.0 wt% **BCP1** material was brittle: it exhibited high flexural strength and modulus combined with low fracture toughness. The incorporation of **BCP1** did not lead to high fracture toughness values. This result can probably be explained by the crosslink density differences. It was indeed demonstrated that the crosslink density plays a significant role in the BCP’s toughening ability. As an example, the addition of BCP to dimethacrylate mixtures is an inefficient toughening strategy [15]. Moreover, concerning the nature of the urethane macromonomers, the following trend was identified: the longer the spacer, the higher the fracture toughness [19]. The presence of four methacrylate groups (instead of two) clearly increases the crosslink density of the cured material, which suppresses the efficiency of the toughening. The replacement of the dimethacrylate macromonomer **UM1** with the corresponding diacrylate **UM8** was also shown to be disadvantageous. A lower T_g_ was obtained, which probably results in a weakening of the material. Indeed, lower flexural strength and modulus values were obtained. The increased flexibility of the material did not lead to higher fracture toughness values. K_max_ and W_f_ were even lower than for the **UM1**-based material.

## 5. Conclusions

The nature of the synthesized urethane macromonomers was shown to have a strong influence on the mechanical properties of light-cured **BCP1**-based materials. **UM1** and **UM2** were identified to be the most interesting compounds. Indeed, the combination of those macromonomers with OMIMA and the right amount of **BCP1** successfully enabled the formulation of light-cured high-impact denture bases. DLP 3D printing is a multi-step process. The mechanical properties of printed materials can be affected by several process parameters (e.g., amount/nature of the photoinitiator, layer thickness, nature of the cleaning and post-processing steps, etc.). The next step of this work will be to print the **UM1**- and **UM2**-based formulations and to optimize the parameters in order to obtain similar mechanical properties to bulk-cured materials.

## Figures and Tables

**Table 1:** Viscosity of **BCP1**-based **UMs1-6**/OMIMA and **DMA1**/OMIMA formulations as well as T_g_ and DBC of the corresponding light-cured materials.

Resin	Viscosity (Pa s)	DBC (%)	T_g_ (°C)
**UM1**/OMIMA (1/1: wt/wt) + 5.0 wt% **BCP1**	3.38	100	84
**UM2**/OMIMA (1/1: wt/wt) + 5.0 wt% **BCP1**	6.09	100	78
**UM3**/OMIMA (1/1: wt/wt) + 5.0 wt% **BCP1**	3.07	100	72
**UM4**/OMIMA (1/1: wt/wt) + 5.0 wt% **BCP1**	3.12	100	79
**UM5**/OMIMA (1/1: wt/wt) + 5.0 wt% **BCP1**	6.60	100	89
**UM6**/OMIMA (1/1: wt/wt) + 5.0 wt% **BCP1**	8.02	100	87
**DMA1**/OMIMA (1/1: wt/wt) + 5.0 wt% **BCP1**	2.27	100	101

**Table 2:** Flexural strength and flexural modulus of light-cured **UM1**/OMIMA, **UM2**/OMIMA, **UM3**/OMIMA, and **DMA1**/OMIMA materials containing various amounts of **BCP1**.

Resin	BCP1 (wt%)	Flexural Strength (MPa)	Flexural Modulus (MPa)
**UM1**/OMIMA (1/1: wt/wt)	5.0	65.9 ± 1.0	2037 ± 60
**UM1**/OMIMA (1/1: wt/wt)	4.0	70.1 ± 2.0	2171 ± 56
**UM2**/OMIMA (1/1: wt/wt)	5.0	62.1 ± 3.8	1872 ± 140
**UM2**/OMIMA (1/1: wt/wt)	4.0	72.0 ± 1.9	2250 ± 80
**UM3**/OMIMA (1/1: wt/wt)	5.0	58.5 ± 1.9	1861 ± 70
**UM3**/OMIMA (1/1: wt/wt)	4.0	60.7 ± 1.5	1857 ± 57
**UM3**/OMIMA (1/1: wt/wt)	3.0	63.9 ± 4.9	1963 ± 216
**DMA1**/OMIMA (1/1: wt/wt)	5.0	75.2 ± 3.0	2166 ± 175
**DMA1**/OMIMA (1/1: wt/wt)	6.0	67.0 ± 1.5	2009 ± 50
**DMA1**/OMIMA (1/1: wt/wt)	7.0	61.6 ± 1.7	1900 ± 106

**Table 3:** Maximum stress intensity factor (K_max_) and total fracture work (W_f_) of light-cured **UM1**/OMIMA, **UM2**/OMIMA, **UM3**/OMIMA, and **DMA1**/OMIMA materials containing various amounts of **BCP1**.

Resin	BCP1 (wt%)	K_max_ (MPa m^1/2^)	W_f_ (J m^−2^)
**UM1**/OMIMA (1/1: wt/wt)	5.0	2.477 ± 0.142	1478 ± 51
**UM1**/OMIMA (1/1: wt/wt)	4.0	2.285 ± 0.123	844 ± 18
**UM2**/OMIMA (1/1: wt/wt)	5.0	2.303 ± 0.124	1695 ± 43
**UM2**/OMIMA (1/1: wt/wt)	4.0	2.531 ± 0.075	963 ± 33
**UM3**/OMIMA (1/1: wt/wt)	5.0	2.466 ± 0.042	1623 ± 19
**UM3**/OMIMA (1/1: wt/wt)	4.0	2.561 ± 0.044	1512 ± 220
**UM3**/OMIMA (1/1: wt/wt)	3.0	2.443 ± 0.086	834 ± 21
**DMA1**/OMIMA (1/1: wt/wt)	5.0	2.318 ± 0.065	781 ± 21
**DMA1**/OMIMA (1/1: wt/wt)	6.0	2.329 ± 0.073	893 ± 43
**DMA1**/OMIMA (1/1: wt/wt)	7.0	2.240 ± 0.090	1264 ± 147

**Table 4:** Viscosity of **BCP1**-based **UM1**/OMIMA and **UMs7-8**/OMIMA formulations as well as T_g_ and DBC of the corresponding light-cured materials.

Resin	Viscosity (Pa s)	DBC (%)	T_g_ (°C)
**UM1**/OMIMA (1/1: wt/wt) + 5.0 wt% **BCP1**	3.38	100	84
**UM7**/OMIMA (1/1: wt/wt) + 5.0 wt% **BCP1**	3.35	93 ± 1	107
**UM8**/OMIMA (1/1: wt/wt) + 5.0 wt% **BCP1**	4.35	100	72

**Table 5:** Flexural strength and flexural modulus of light-cured **UM1**/OMIMA and **UMs7-8**/OMIMA materials containing 5.0 wt% of **BCP1**.

Resin	Flexural Strength (MPa)	Flexural Modulus (MPa)
**UM1**/OMIMA (1/1: wt/wt) + 5.0 wt% **BCP1**	65.9 ± 1.0	2037 ± 60
**UM7**/OMIMA (1/1: wt/wt) + 5.0 wt% **BCP1**	71.2 ± 9.1	2496 ± 125
**UM8**/OMIMA (1/1: wt/wt) + 5.0 wt% **BCP1**	58.8 ± 2.1	1836 ± 114

**Table 6:** Maximum stress intensity factor (K_max_) and total fracture work (W_f_) of light-cured **UM1**/OMIMA and **UMs7-8**/OMIMA materials containing 5.0 wt% of **BCP1**.

Resin	K_max_ (MPa m^1/2^)	W_f_ (J m^−2^)
**UM1**/OMIMA (1/1: wt/wt) + 5.0 wt% **BCP1**	2.477 ± 0.142	1478 ± 51
**UM7**/OMIMA (1/1: wt/wt) + 5.0 wt% **BCP1**	1.081 ± 0.045	135 ± 8
**UM8**/OMIMA (1/1: wt/wt) + 5.0 wt% **BCP1**	2.108 ± 0.028	1161 ± 183

## Data Availability

The data presented in this study are available on request from the corresponding author.

## Supplementary Materials

The following supporting information can be downloaded at: https://www.mdpi.com/article/10.3390/polym17131761/s1.
