# Supplementary material for: Urethane Macromonomers: Key Components for the Development of Light-Cured High-Impact Denture Bases
Source: Polymers (Basel). 2025 Jun 26;17(13):1761. doi: 10.3390/polym17131761 (PMC12252331; doi:10.3390/polym17131761)
Supplement: Supplementary file 1 [file polymers-17-01761-s001.zip › polymers-3673614-supplementary.pdf]

Supplementary information

Urethane Macromonomers: Key Components for the Development of Light-Cured High-Impact Denture Bases

Benjamin Grob [1], Pascal Fässler [1], Iris Lamparth [1], Sadini Omeragic [1], Kai Rist [1], Loïc Vidal [2,3], Jacques Lalevée [2,3] and Yohann Catel [1]

[1] Ivoclar Vivadent AG, Bendererstrasse 2, FL-9494 Schaan, Liechtenstein
[2] Université de Haute-Alsace, CNRS, IS2M UMR 7361, F-68100 Mulhouse, France; jacques.lalevee@uha.fr
[3] Université de Strasbourg, France

1) ¹H NMR analysis of urethane macromonomers UMs2-8

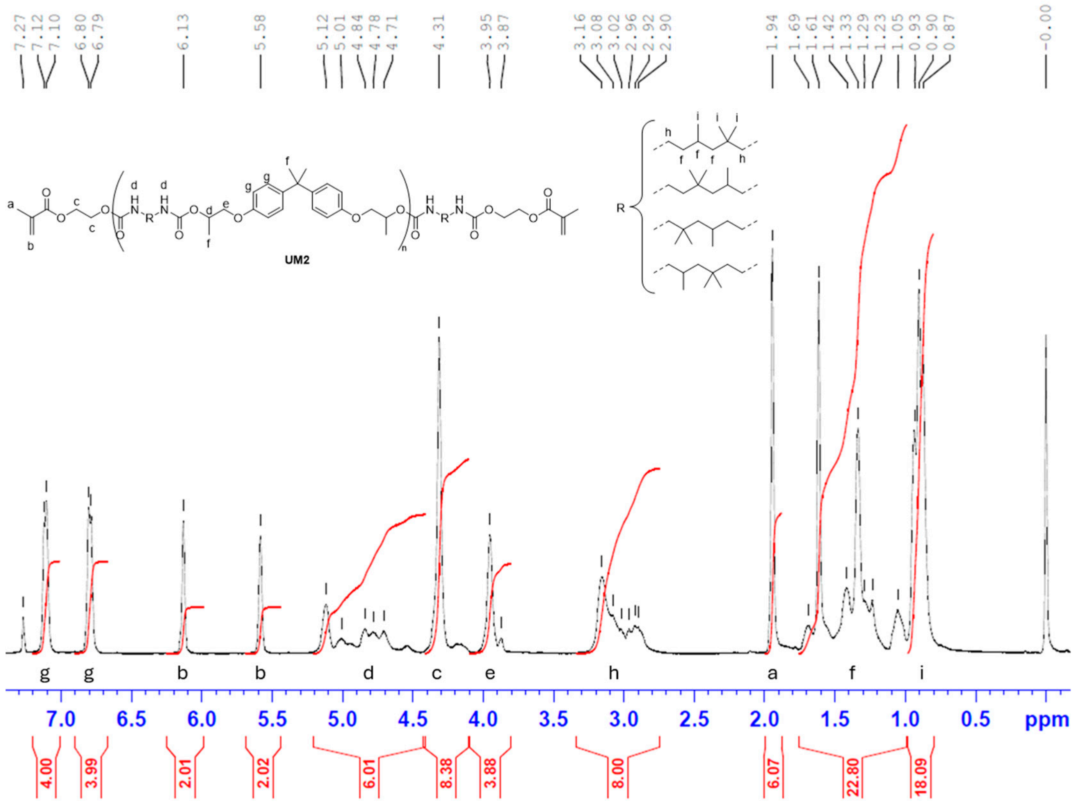

Figure S1. ¹H-NMR spectrum of **UM2**.

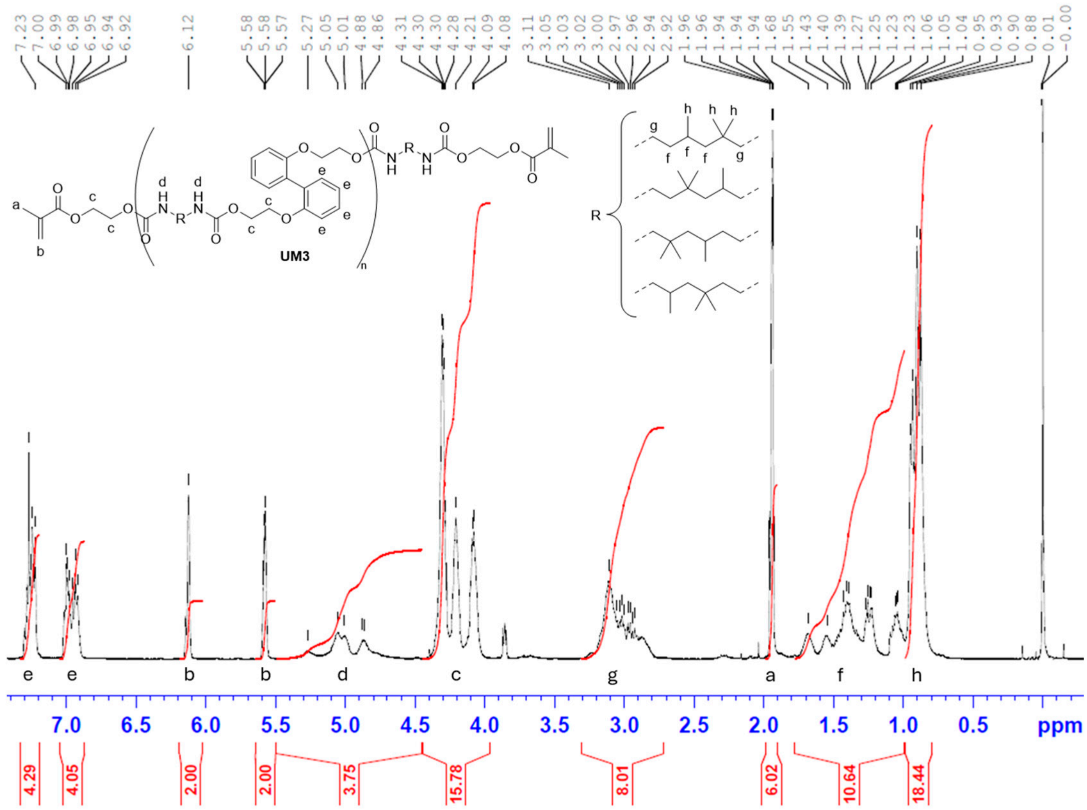

Figure S2. ^{1}H-NMR spectrum of **UM3**.

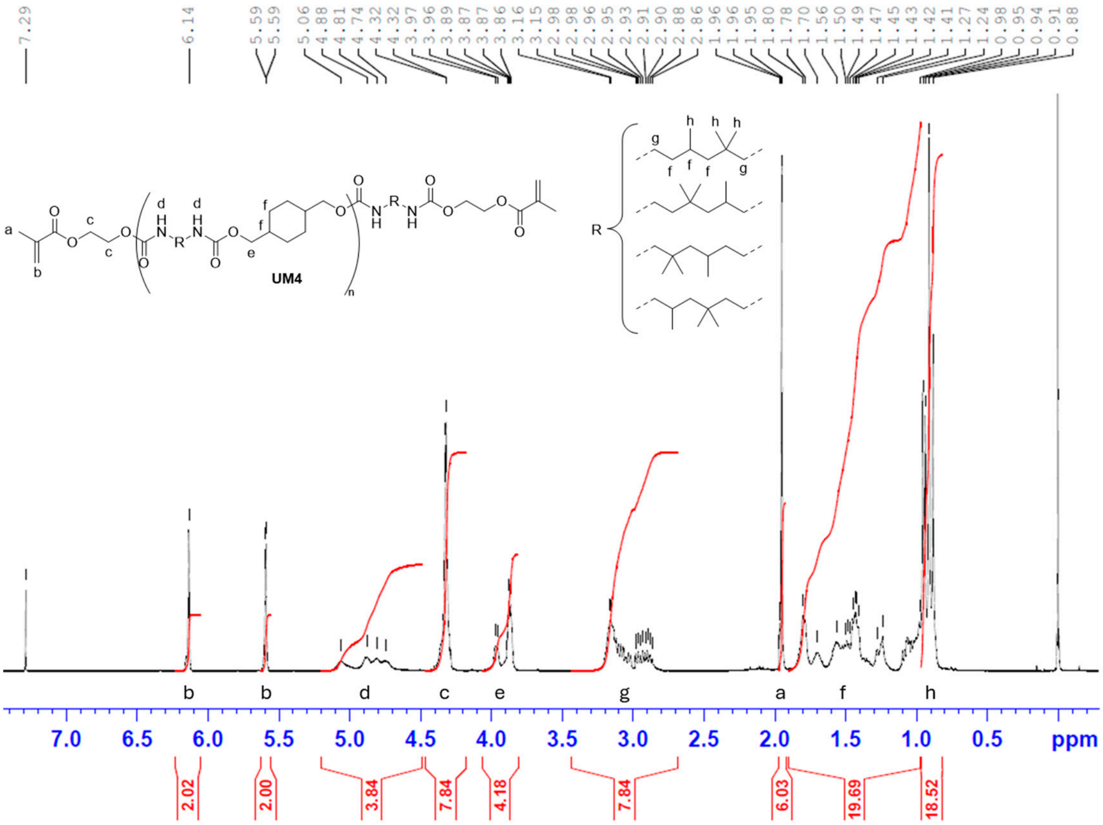

Figure S3. ^{1}H-NMR spectrum of **UM4**.

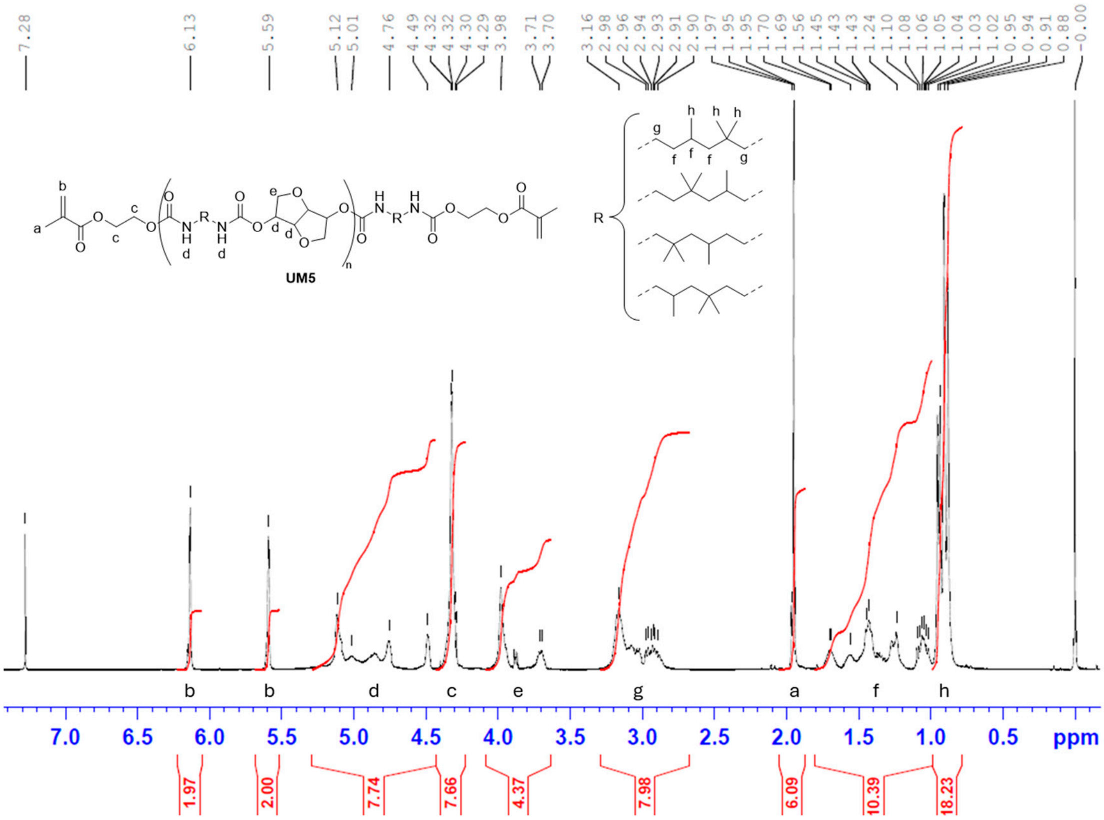

Figure S4. ^{1}H-NMR spectrum of **UM5**.

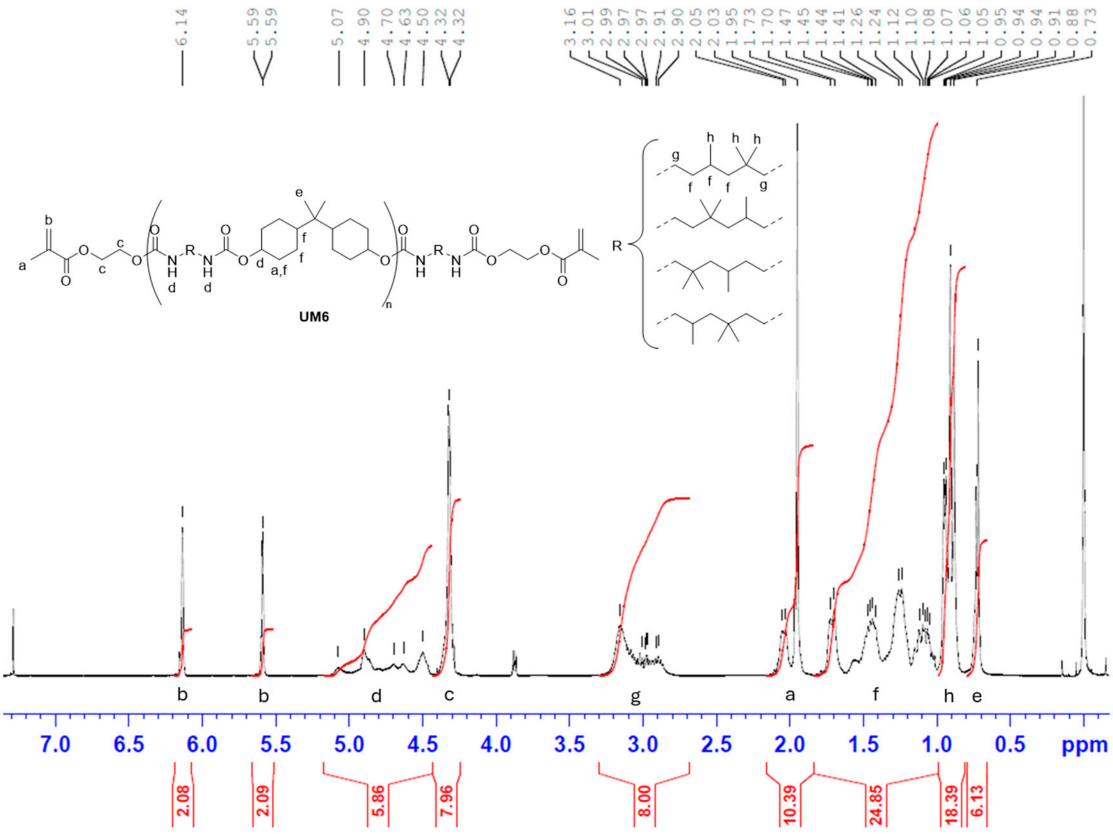

Figure S5. ^{1}H-NMR spectrum of **UM6**.

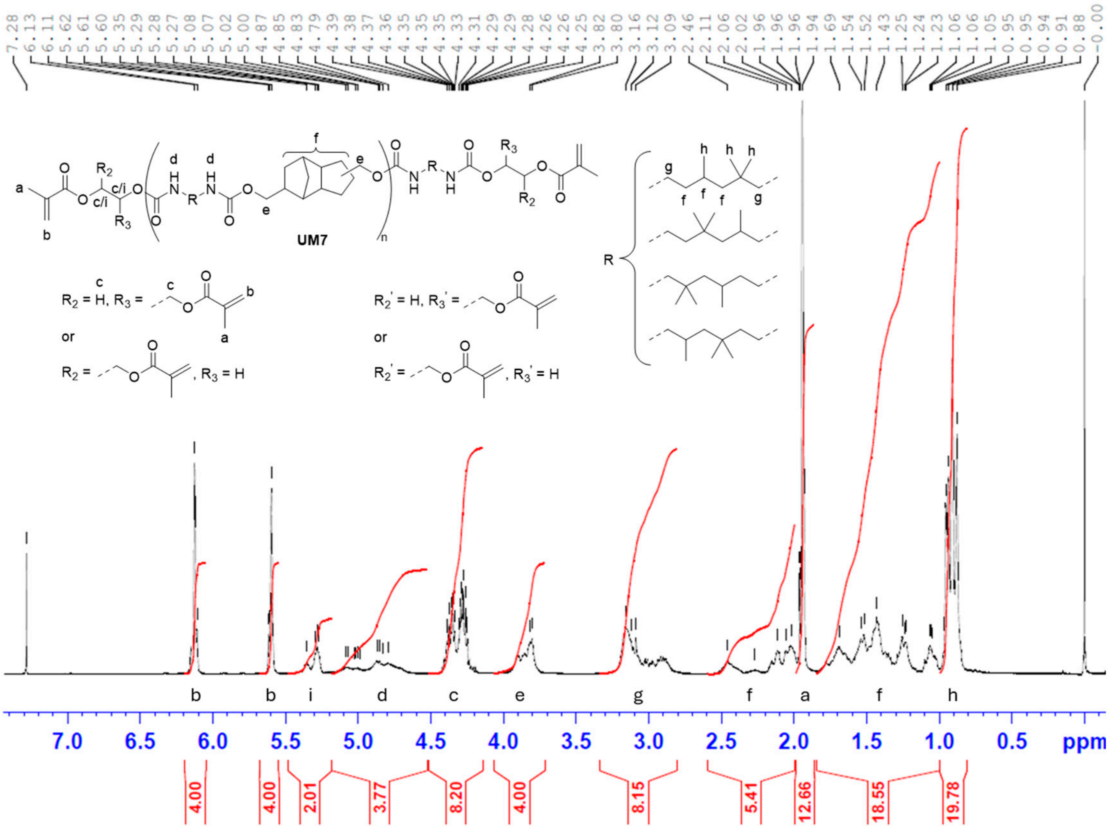

Figure S6. ^{1}H-NMR spectrum of **UM7**.

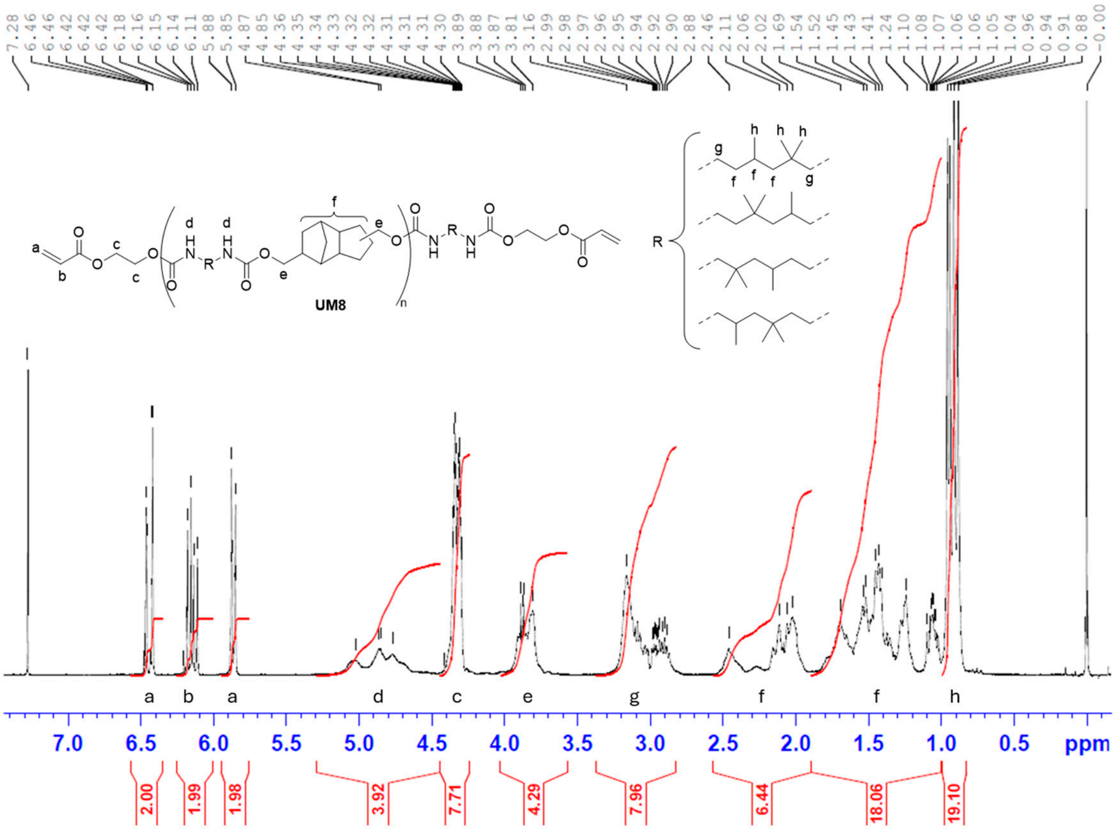

Figure S7. ^{1}H-NMR spectrum of **UM8**.

2) GPC analysis of the urethane macromonomers UMs2-6

GPC measurement was performed on an instrument having four PSS-SDV gel columns (particle size = 5 μm) with porosity range from 10^2 to 10^5 Å (PSS, Mainz, Germany) together with a refractive index detector (Agilent Technologies 1200 Series). THF (HPLC grade) was used as a solvent (for dissolving polymer and as eluting solvent) with a flow rate of 1.0 mL/min. As an internal standard, toluene (HPLC grade) was used. The calibration was performed with narrowly distributed polystyrene (PS) homo-polymers (PSS calibration kit). An injection volume of 20 μL was used for the measurements. The sample was dissolved in THF and filtered through a 0.22 μm PTFE filter before analysis.

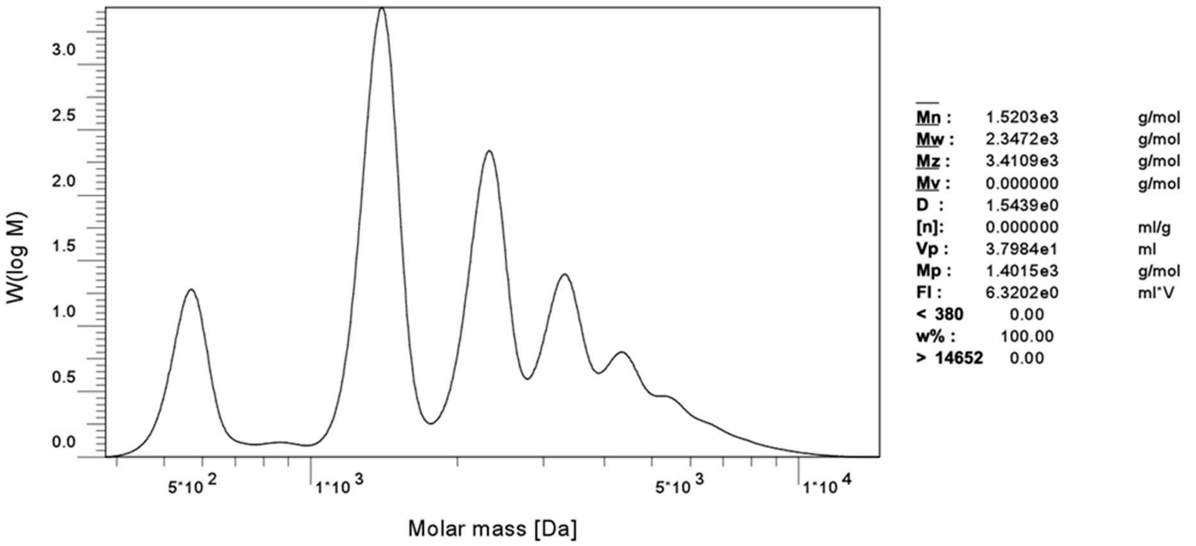

Figure S8. GPC measurement of compound **UM2**.

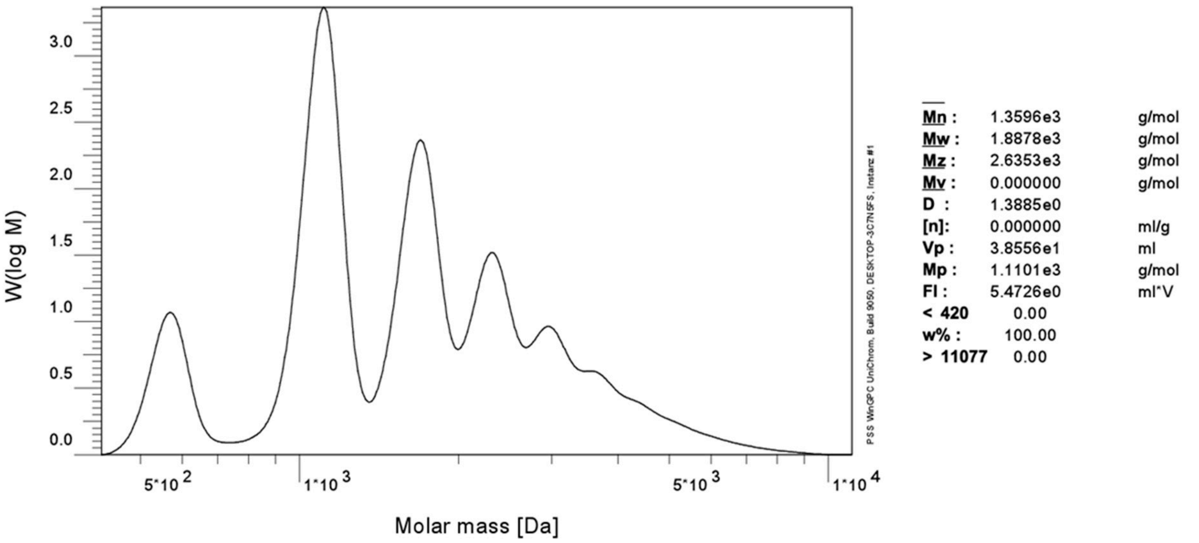

Figure S9. GPC measurement of compound **UM3**.

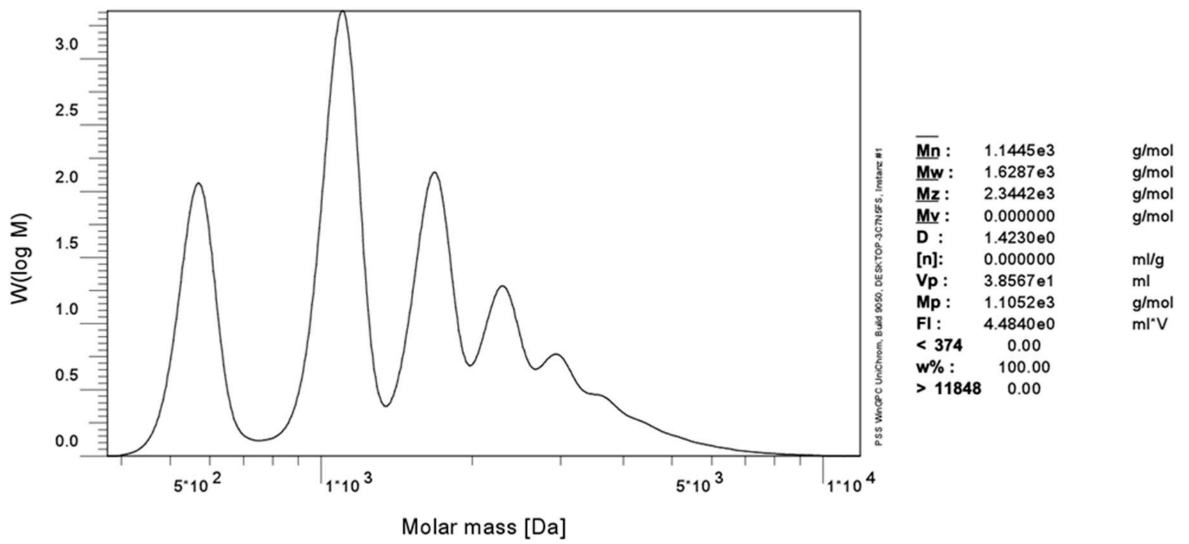

Figure S10. GPC measurement of compound **UM4**.

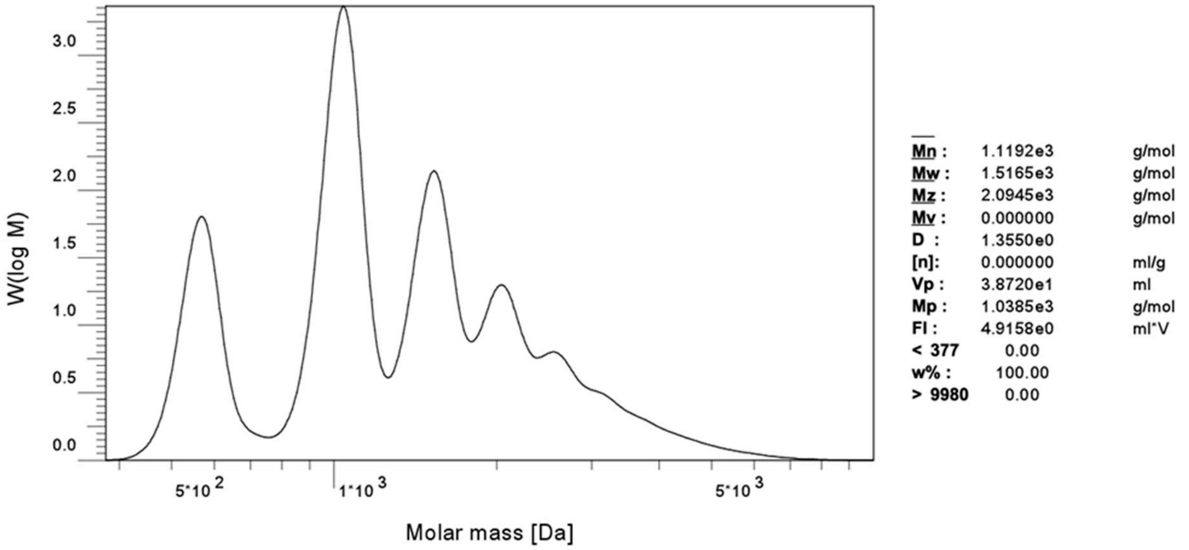

Figure S11. GPC measurement of compound **UM5**.

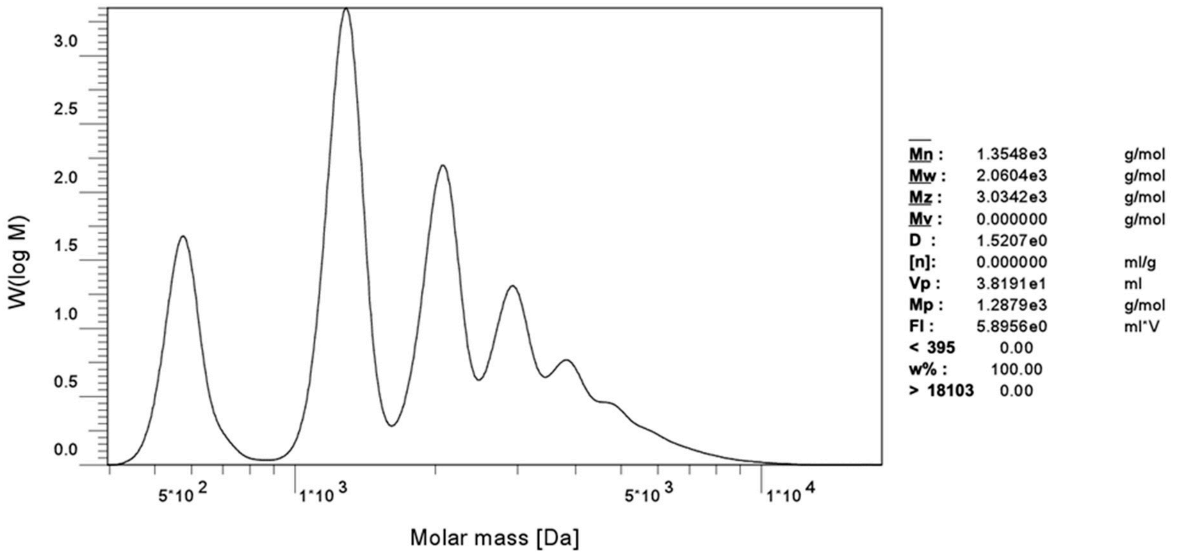

Figure S12. GPC measurement of compound **UM6**.

3) NIR analysis of the cured and uncured formulations

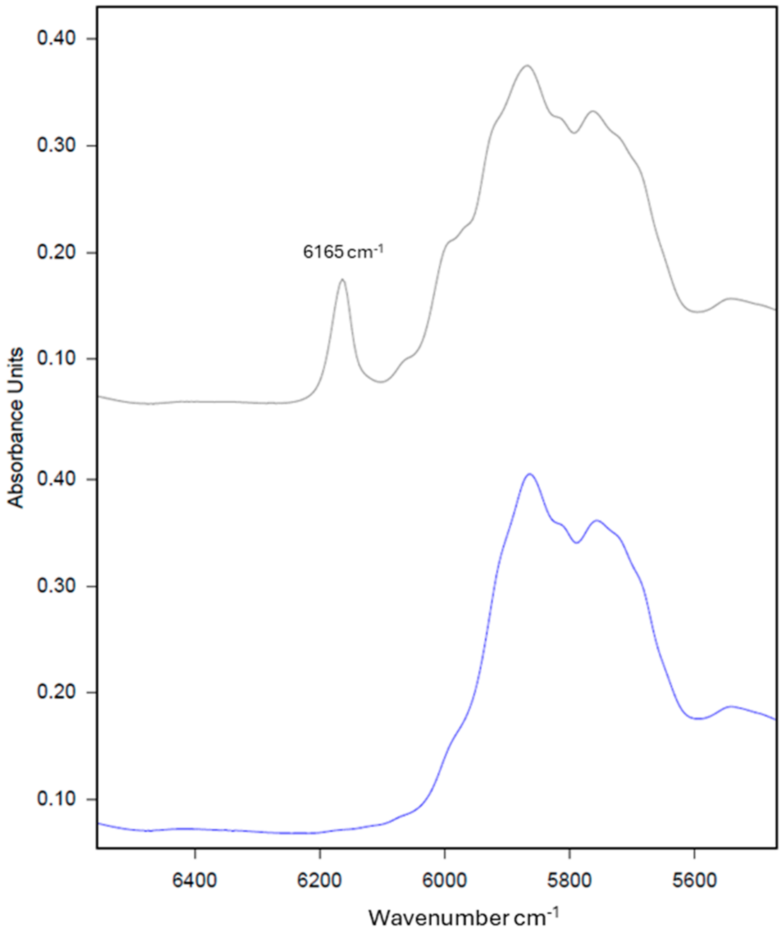

Figure S13. NIR measurement of the uncured (top) and cured monomer mixture **UM2**/OMIMA (1/1: wt/wt) + 5.0 wt% **BCP1** (bottom).

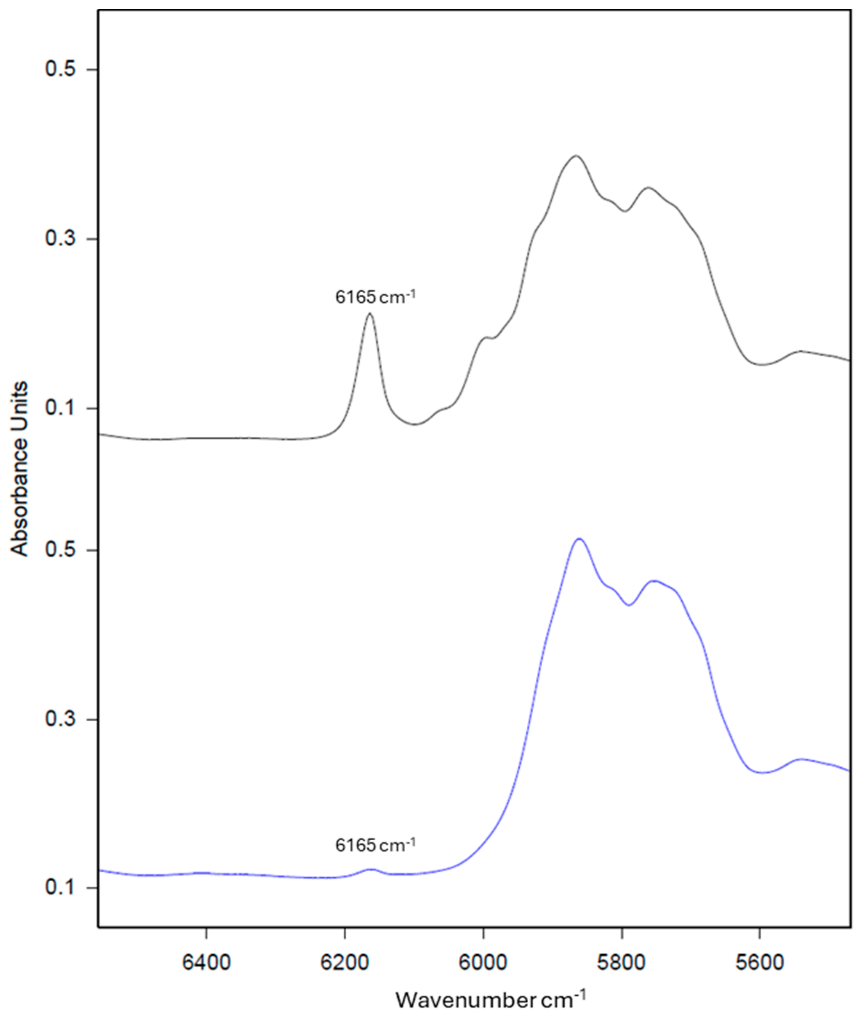

Figure S14. NIR measurement of the uncured (top) and cured monomer mixture **UM7**/OMIMA (1/1: wt/wt) + 5.0 wt% **BCP1** (bottom).

4) DMTA analysis

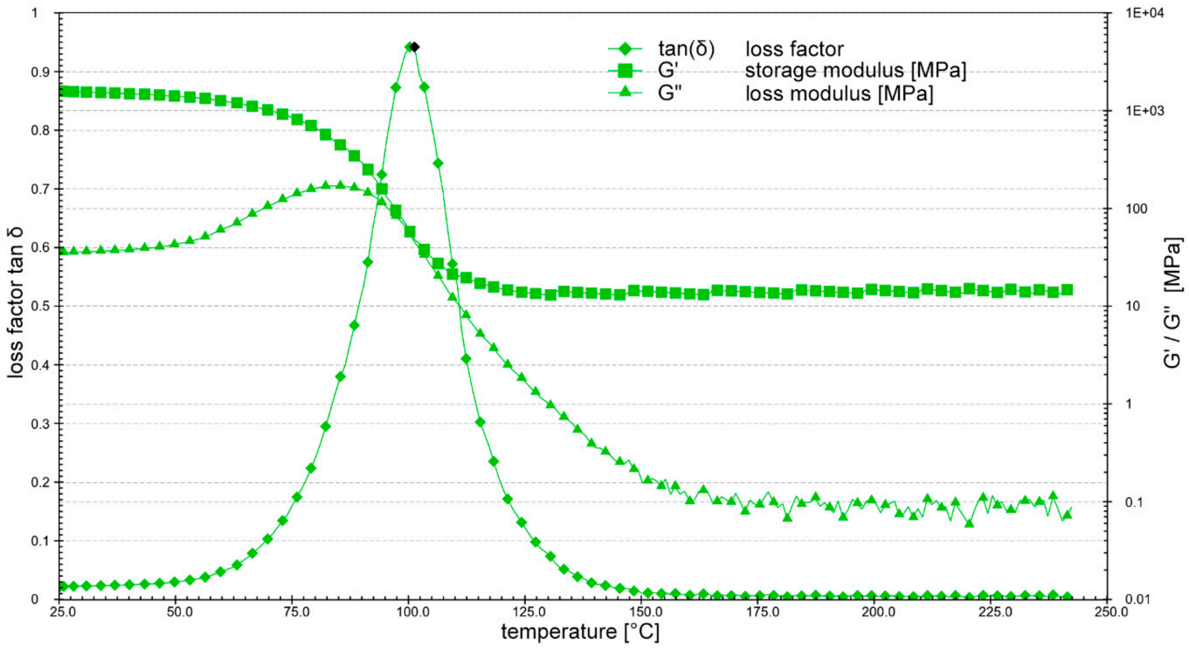

Figure S15. Storage modulus, loss modulus, and tan(δ) for the light-cured **DMA1**/OMIMA (1/1: wt/wt) + 5.0 wt% **BCP1** material.

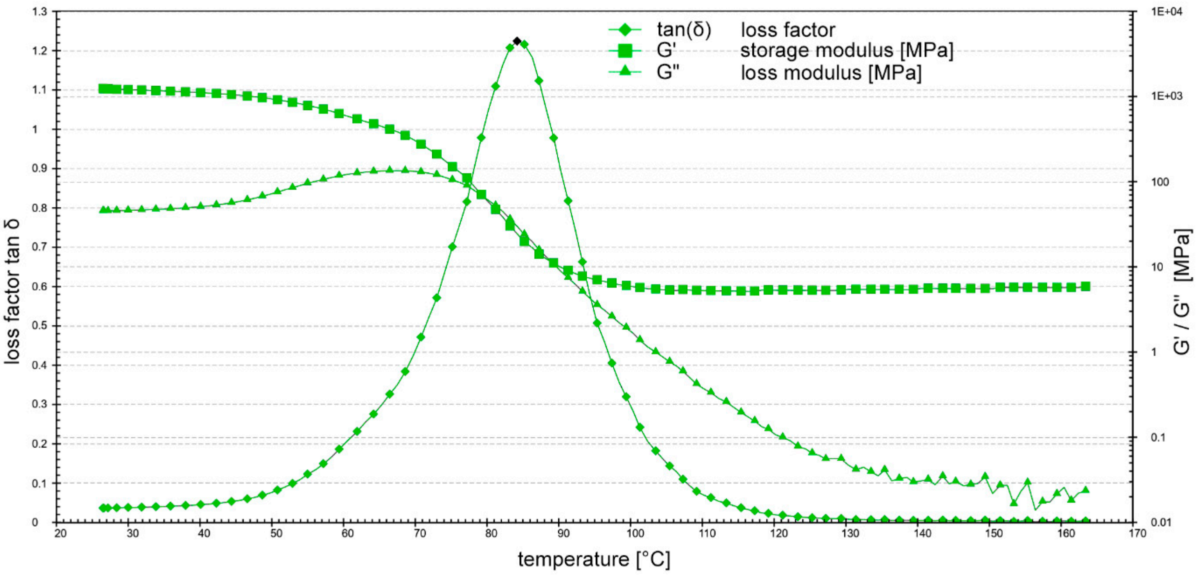

Figure S16. Storage modulus, loss modulus, and tan(δ) for the light-cured **UM1**/OMIMA (1/1: wt/wt) + 5.0 wt% **BCP1** material.

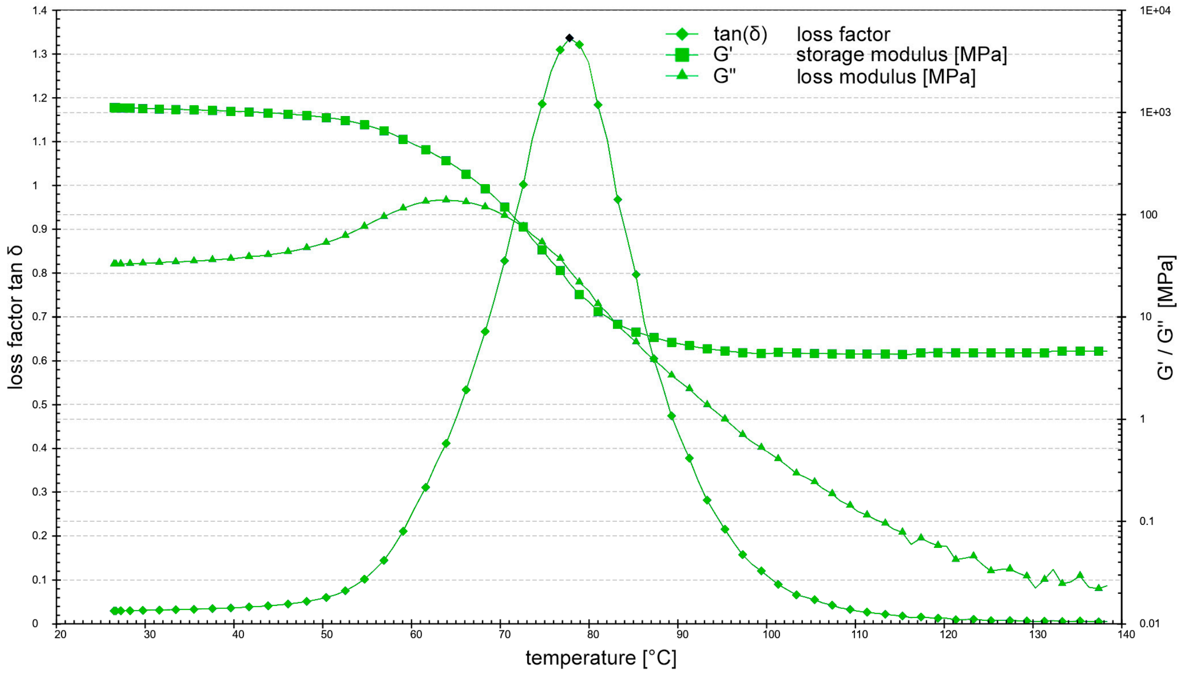

Figure S17. Storage modulus, loss modulus, and tan(δ) for the light-cured **UM2**/OMIMA (1/1: wt/wt) + 5.0 wt% **BCP1** material.

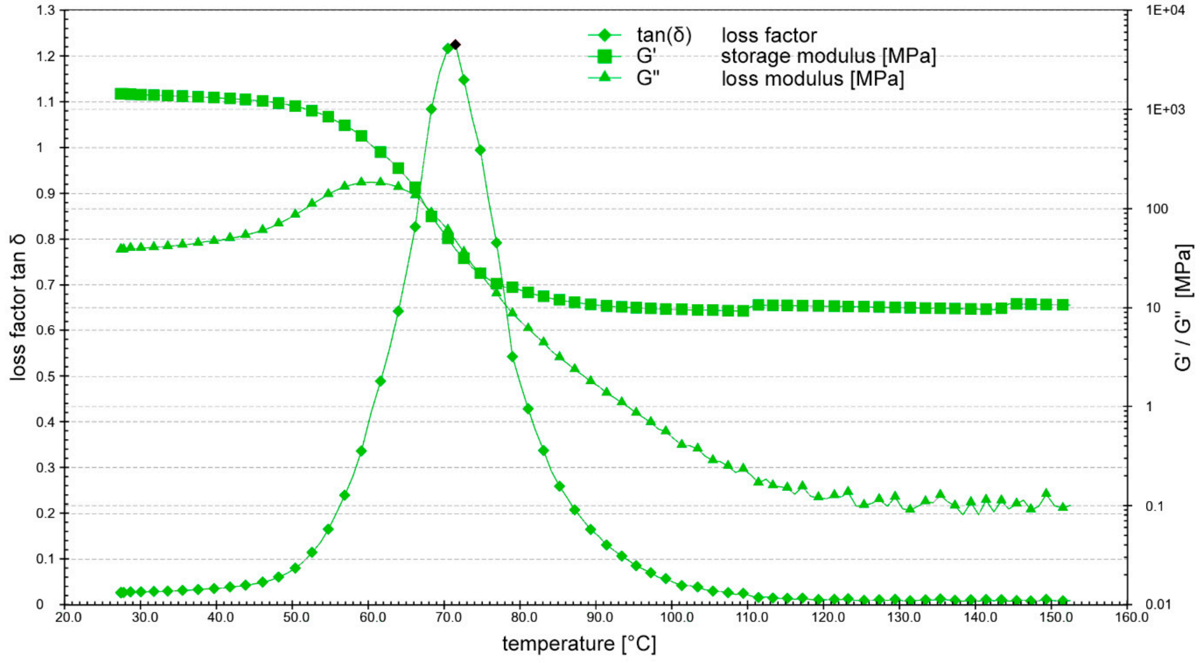

Figure S18. Storage modulus, loss modulus, and tan(δ) for the light-cured **UM3**/OMIMA (1/1: wt/wt) + 5.0 wt% **BCP1** material.

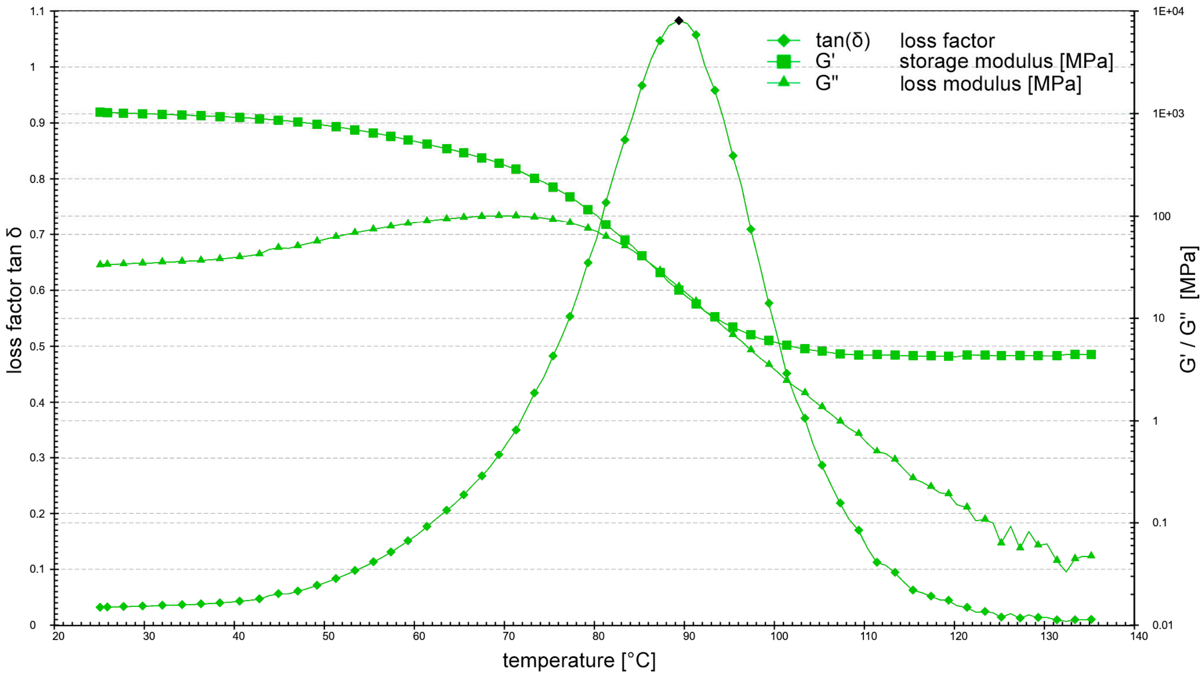

Figure S19. Storage modulus, loss modulus, and tan(δ) for the light-cured **UM4**/OMIMA (1/1: wt/wt) + 5.0 wt% **BCP1** material.

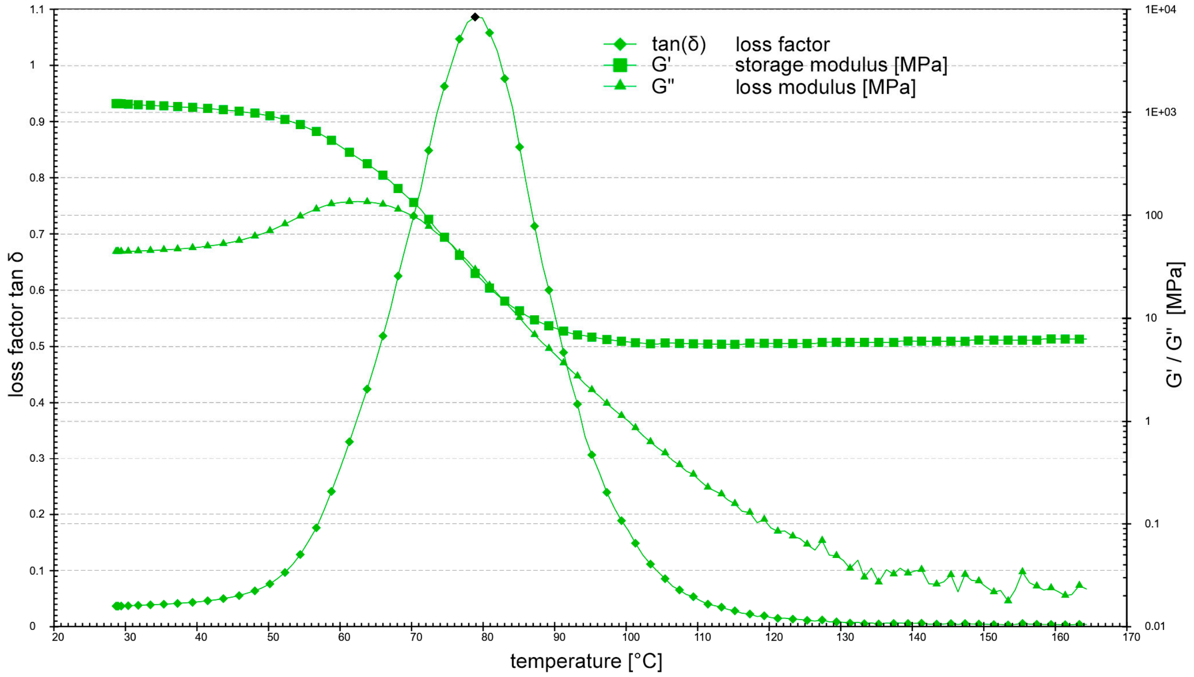

Figure S20. Storage modulus, loss modulus, and tan(δ) for the light-cured **UM5**/OMIMA (1/1: wt/wt) + 5.0 wt% **BCP1** material.

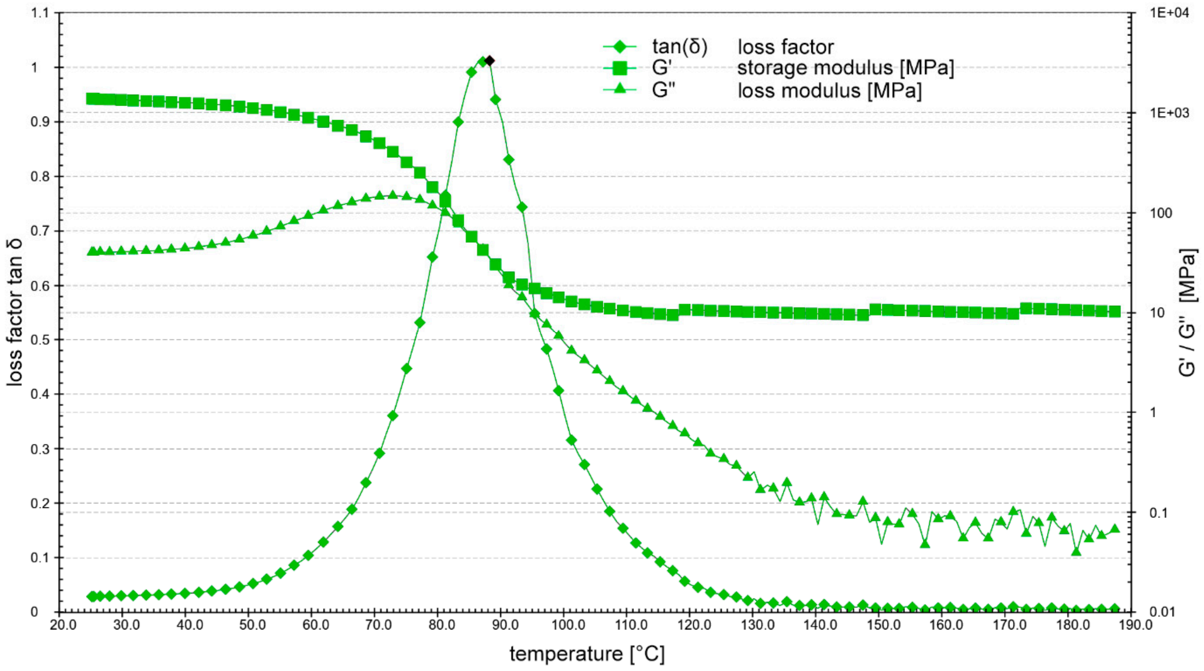

Figure S21. Storage modulus, loss modulus, and tan(δ) for the light-cured **UM6**/OMIMA (1/1: wt/wt) + 5.0 wt% **BCP1** material.

5) Stress/strain curve

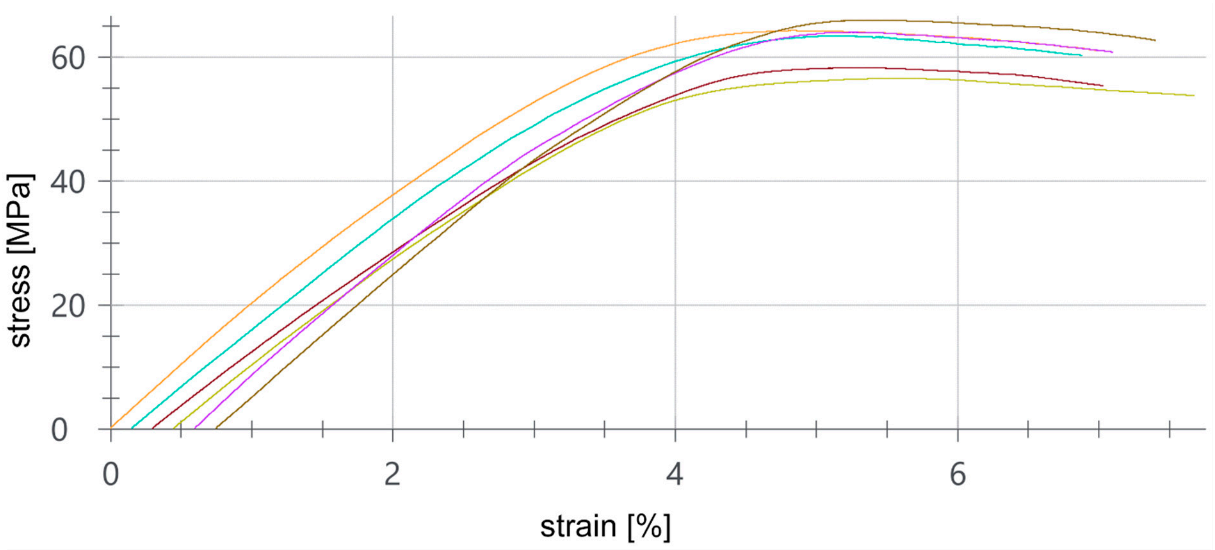

Figure S22. Stress/strain curve for the light-cured **UM2**/OMIMA (1/1: wt/wt) + 5.0 wt% **BCP1** material.
